# Shaping Magnetic Hyperthermia Properties through Nanoparticle Surface‐Ligand Design: Implications for Cellular Responses

**DOI:** 10.1002/smll.202507665

**Published:** 2025-10-25

**Authors:** Lukas Hertle, Alberto López‐Ortega, Hao Ye, Alba Martínez‐Jiménez de Allo, Eneko Garaio, Valentin Gantenbein, Joaquim Llacer‐Wintle, Ishika Paul, Sarina Nigg, Josep Puigmartí‐Luis, Marta Estrader, Xiang‐Zhong Chen, Bradley J. Nelson, Salvador Pané

**Affiliations:** ^1^ Multi‐Scale Robotics Lab Institute of Robotics and Intelligent Systems ETH Zürich, Tannenstrasse 3 Zürich CH‐8092 Switzerland; ^2^ Departamento de Ciencias Campus de Arrosadía Universidad Pública de Navarra Pamplona 31006 Spain; ^3^ Institute for Advanced Materials and Mathematics (INAMAT^2^) Universidad Pública de Navarra Pamplona E‐31006 Spain; ^4^ Departament de Ciència dels Materials i Química Física Institut de Química Teòrica i Computacional University of Barcelona Martí i Franquès, 1 Barcelona 08028 Spain; ^5^ Institució Catalana de Recerca i Estudis Avançats (ICREA) Pg. Lluís Companys 23 Barcelona 08010 Spain; ^6^ Department of Inorganic and Organic Chemistry Inorganic Chemistry Section University of Barcelona Carrer de Martí i Franquès, 1‐11 Barcelona 08028 Spain; ^7^ Institute of Nanoscience and Nanotechnology (IN2UB) University of Barcelona Carrer de Martí i Franquès, 1‐11 Barcelona 08028 Spain; ^8^ International Institute of Intelligent Nanorobots and Nanosystems College of Intelligent Robotics and Advanced Manufacturing State Key Laboratory of Photovoltaic Science and Technology Shanghai Frontiers Science Research Base of Intelligent Optoelectronics and Perception Institute of Optoelectronics Fudan University Shanghai 200433 China; ^9^ Zhejiang Key Laboratory of Extreme Environment Functional Materials Yiwu Research Institute of Fudan University Yiwu 322000 China

**Keywords:** iron oxide, ligands, magnetic hyperthermia, magnetic nanoparticles, surface modification

## Abstract

Magnetic iron oxide nanoparticles have attracted increasing attention for their potential use in biomedicine over the last few decades. Their inherent characteristics have enabled novel therapeutic approaches such as magnetic hyperthermia. To maximize the therapeutic efficacy, several research efforts have been focused on the optimization of these nanoparticles in terms of their size, morphology, and crystal structure etc. However, no consensus has been reached regarding the optimal surface design. To gain deeper insight into this complex phenomenon, the influence of a variety of surface ligands on the magnetic, hyperthermic, and colloidal behaviors of the magnetic iron oxide nanoparticles, along with their influence on cellular viability, is investigated. The results revealed that the molecular structure of the ligands, including both the anchoring group and molecular chain, plays a critical role in determining the above‐mentioned properties and performance. This work lays the groundwork for surface engineering of magnetic nanoparticles, emphasizing the need to consider the magneto‐hyperthermic performance, colloidal stabilities, and the cellular interactions as interconnected factors that critically influence their clinical applicability.

## Graphical abstract

Magnetic iron oxide nanoparticles are functionalized with several ligands varying anchoring groups, chain length, and architecture, and terminal functionality. Aggregation and colloidal stability are quantified. Field‐dependent magnetization and specific absorption rate are measured to assess dipolar coupling and surface anisotropy effect. Cellular viability is investigated. Together, these measurements map how surface chemistry connects aggregation, magnetization dynamics, hyperthermic heating, and cytocompatibility.

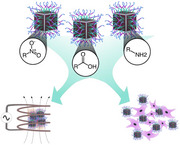

## 1 Introduction

Magnetic iron oxide nanoparticles (MIONPs) hold great promise for revolutionizing various biomedical applications due to their favorable magnetic properties, excellent biocompatibility, and versatile surface design options.^[^ ^1^, ^2^, ^3^, ^4^, ^5^, ^6^ ^]^ Their potential applications span targeted drug delivery,^[^ ^7^, ^8^ ^]^ medical imaging enhancement,^[^ ^9^, ^10^, ^11^, ^12^, ^13^ ^]^ and the clinical translation of experimental therapies such as magnetic hyperthermia (MHT).^[^ ^14^, ^15^, ^16^, ^17^ ^]^ While currently not widely adapted, MHT was reported to significantly increase the efficacy of conventional cancer therapies, including chemotherapy and radiotherapy.^[^ ^18^ ^]^ A grand challenge hindering the clinical translation of MHT lies in the design of particles capable of reliably and efficiently generating heat when exposed to alternating magnetic fields at medically safe frequencies. The heating capability of MIONPs is characterized by their specific absorption rate (*SAR*). In the context of MHT, the *SAR* refers to the power absorbed by the MIONPs from the alternating magnetic field, and it is normalized by the mass or magnetic material (units: W g^−1^ or watts per gram of MIONPs). The particle specific *SAR* is thereby commonly described according to^[^ ^19^ ^]^

(1) $$SAR=-f\mu_{0}\int_{0}^{1/f}M_{t}dH$$

where *M_t_* is the mass‐normalized dynamic magnetization, μ_0_ is the permeability of free space, while *f* and *H* are referring to the applied alternating magnetic field frequency and intensity, respectively. From the above stated formula, it can be seen that the MIONPs *SAR* is proportional to the alternating magnetic field frequency (*f*) and to the area of the magnetic AC hysteresis loop, which, in an *M*_t_‐*H* graph, corresponds to the area enclosed by the mass‐normalized dynamic magnetization under the alternating magnetic field.^[^ ^16^ ^]^

Extensive experimental investigations throughout the past years have shown that the observed *SAR* values depend on a range of MIONP properties, including their size and morphology,^[^ ^20^, ^21^ ^]^ polydispersity,^[^ ^22^ ^]^ crystal structure,^[^ ^23^, ^24^ ^]^ aggregation state, particle concentration, and colloidal stability.^[^ ^25^, ^26^, ^27^, ^28^ ^]^ This multi‐variant dependence presents a major challenge to the rational design of MIONPs capable of producing efficient and reproducible MHT. Considering that the field frequencies are predetermined by medical safety standards, and particle concentrations should stay below cytotoxic thresholds,^[^ ^29^, ^30^, ^31^, ^32^, ^33^ ^]^ optimizing the magnetic hysteresis area of the MIONPs becomes critical. The magnetization of MIONPs is governed by several factors, with magnetic anisotropy, originating from magneto‐crystallinity, surface, and shape contributions, being one of the most important. To understand the contributions of each, it is essential to employ model systems comprising MIONPs with well‐defined crystalline structure, size, and morphology. Consequently, the extensive research efforts have focused on the synthesis of such particles with highly defined shapes and properties across a wide size range.^[^ ^34^, ^35^, ^36^, ^37^, ^38^, ^39^, ^40^, ^41^, ^42^ ^]^

Despite these efforts, the clinical translation of MHT still remains challenging, as a wide variability in magnetic hyperthermia properties continues to be reported, consequently hindering both the reproducibility and selection of the appropriate magnetic nano‐objects. Among the multi‐fold factors, the impact of dissimilar ligand molecules, whose structure (anchoring‐ and end‐groups) can significantly alter the particles’ magnetic and colloidal behavior, cannot be neglected. For instance, different particle aggregation states induced by the ligand can change the magnetic dipolar interactions between particles, which ultimately affect their magnetic hyperthermia performances.^[^ ^25^ ^]^ A detailed analysis of the surface electron orbit structure of MIONPs further unveiled a significant influence of the anchoring groups of the used ligand molecules,^[^ ^43^ ^]^ which determines the particle surface anisotropy, and, consequently, their heating capacity. While these findings have advanced our understanding of the magnetic properties of MIONPs, comprehensive investigations into how surface functionalization affects magnetic, hyperthermic, and cellular responses are still lacking.

In this work, we investigate the impact of different ligands on the magnetic, colloidal, and cytotoxic response of MIONPs by varying the molecular structure, including the anchoring moiety and the chain structure. Interest is placed on establishing reliable correlations between magnetic properties and hyperthermic behavior across different surface chemistries. We further assessed the cell viability of these particles, which revealed a clear terminal moiety dependent response. The presented study is hence aimed to provide additional insights into the occurring surface related phenomena both in terms of ligand‐particle as well as ligand‐media interactions for future MHT applications. Insights into how the surface molecular structure affects particle behavior enable a more effective design strategy for MIONPs in clinical magnetothermal applications.

## 2 Results and Discussion

**Figure 1:**
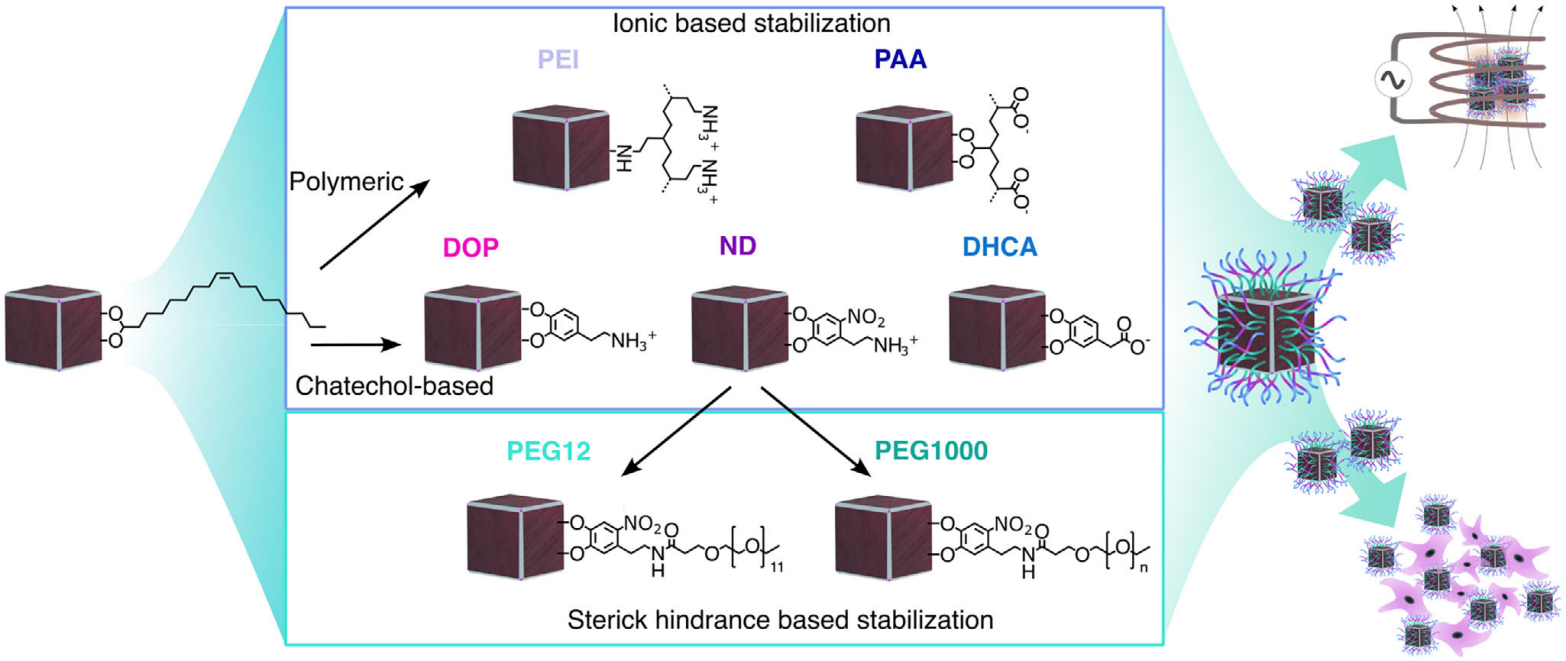
Schematic illustration of undertaken MIONP surface modifications (polyethylenimine (PEI), polyacrylic acid (PAA), dopamine (DOP), 6‐nitrodopamine (ND), 3,4‐dihydroxyhydrocinnamic acid (DHCA), polyethylene glycol 12 coupled to ND (PEG12), and polyethylene glycol (MW:1000) coupled to ND (PEG1000)) and their follow‐up investigations.

To better understand the surface properties influencing the suitability of MIONPs for MHT, we conducted magnetic hyperthermic heating tests and cellular viability assessments of MIONPs functionalized with a variety of ligands. As depicted in **Figure** **1**, we categorized the surface modifications into two groups: ionic stabilization and steric hindrance‐based stabilization. For ionically stabilization, we examined two sub‐types: polymer‐based and catechol‐based ones.

**Figure 2:**
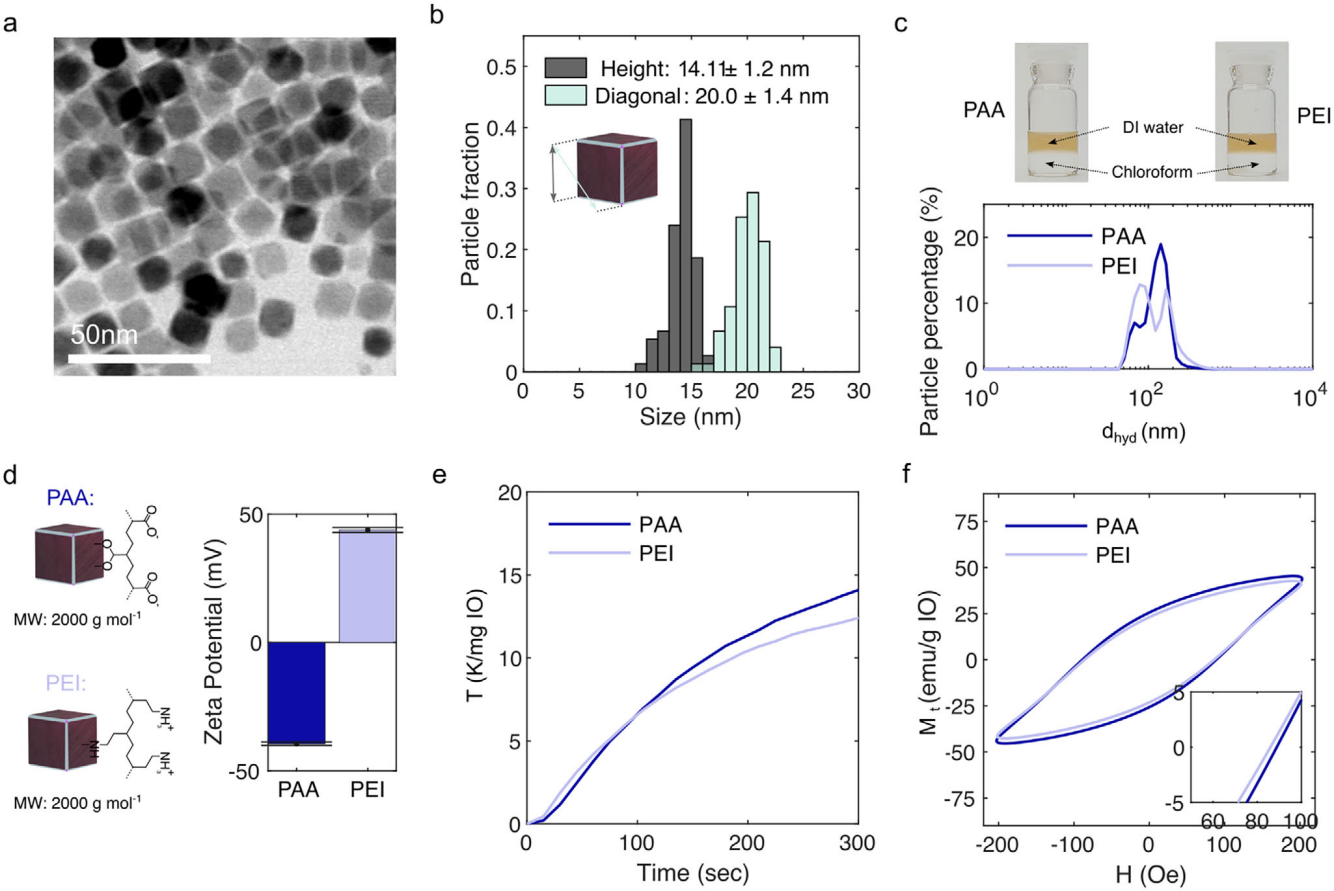
a) A TEM bright‐field image of as‐synthesized OA‐capped MIONPs. b) Size distribution chart of OA‐capped MIONPs statistically derived from TEM images. c) MIONPs in water after PAA and PEI ligand exchanges on top of chloroform, and their hydrodynamic diameters in de‐ionized water. d) Schematic illustration of particle surface ionization and their measured ζ‐potentials in DI water. e) Recorded temperature change of colloidal MIONP suspensions under 480 kHz and 200 Oe AC magnetic field, measured by fiber optic thermometry (Neoptix). f) AC‐hysteresis loops recorded at 480 kHz and 200 Oe of field amplitude.

Ionic stabilization via polymeric surface modifications. We first synthesized monodispersed MIONPs by thermal decomposition of iron(III) acetylacetonate in a mixture of organic solvents with high boiling points and using oleic acid (OA) as a surfactant, following a previously described protocol.^[^ ^44^ ^]^ Transmission electron microscopy (TEM) images (**Figures** **2**a and S1a–d, Supporting Information), revealed the formation of highly uniform cubic‐shaped particles, with an average particle size of 14 ± 1 nm in height and 20 ± 1 nm in diagonal length, as illustrated in Figure 2b. It should be noted that these dimensions were obtained from 2D TEM images. An estimated average particle volume of 2900 ± 600 nm^3^ and surface area of 1200 ± 200 nm^2^ got calculated, yielding an inherent particle surface‐to‐volume ratio of 0.42 ± 0.03 nm^−1^, a relatively high value indicating that a significant fraction of atoms reside at the surface, thereby enhancing reactivity and functionalization efficiency. To provide the as‐synthesized particles with hydrophilicity, we next undertook ligand exchange by replacing OA with two different types of hydrophilic polymers, i.e., polyacrylic acid (PAA) and polyethylenimine (PEI). Both modifications have similar molecular weights but resulted in particles having different anchoring groups on their surface and different induced surface charges. Successful ligand exchange was confirmed by FTIR analysis of the dried MIONPs powders, which most notably showed the vanishing of the OA characteristic C‐H related vibration peaks at 2854 and 2922 cm^−1^, with a simultaneous occurring of the respective hydrophilic ligand molecule characteristic peak patterns (Figure S2, Supporting Information). Both ligand‐exchange procedures, moreover, resulted in the formation of highly stable colloidal MIONP dispersions in de‐ionized (DI) water, which were characterized by a similar hydrodynamic diameter (*d_hyd_*) of ≈128 nm (PAA: *d_hyd_* = 127 ± 54 nm; PEI *d_hyd_* = 128 ± 42 nm), which got obtained by dynamic light scattering (DLS) (Figure 2c). ζ‐potential measurements on the modified particles revealed a positive ζ‐potentials for PEI modified particles (≈+44 mV), which can be associated with the amine groups present on the surface of the particles. In contrast, carboxylic acid‐functionalization via PAA, resulted in a negative ζ‐potential value having a similar magnitude (≈–39 mV) (Figure 2d).

To analyze the impact of the ligands on the MHT performance of the particles, we next performed magnetic heating experiments by exposing diluted MIONP dispersions (≈1.5 mg_IO_ ml^−1^) to an AC magnetic field of 200 Oe operating at a frequency of 480 kHz. As can be seen in Figure 2e, the heating profiles of the colloidal MIONP suspensions reveal variations in their capacity to generate heat, pointing toward a different magnetic relaxation behavior between the samples in dependency of the surface modification. Magnetic AC hysteresis curves measured at the same frequency and field amplitude (Figure 2f and Figure S3, Supporting Information) revealed a modestly increased hysteresis area of 12% for PAA‐modified particles, with *SAR* values of 566 W g^−1^_IO_ and 631 W g^−1^_IO_ for PEI‐ and PAA‐functionalized MIONPs, respectively, being in line with the observed heating rates.

**Figure 3:**
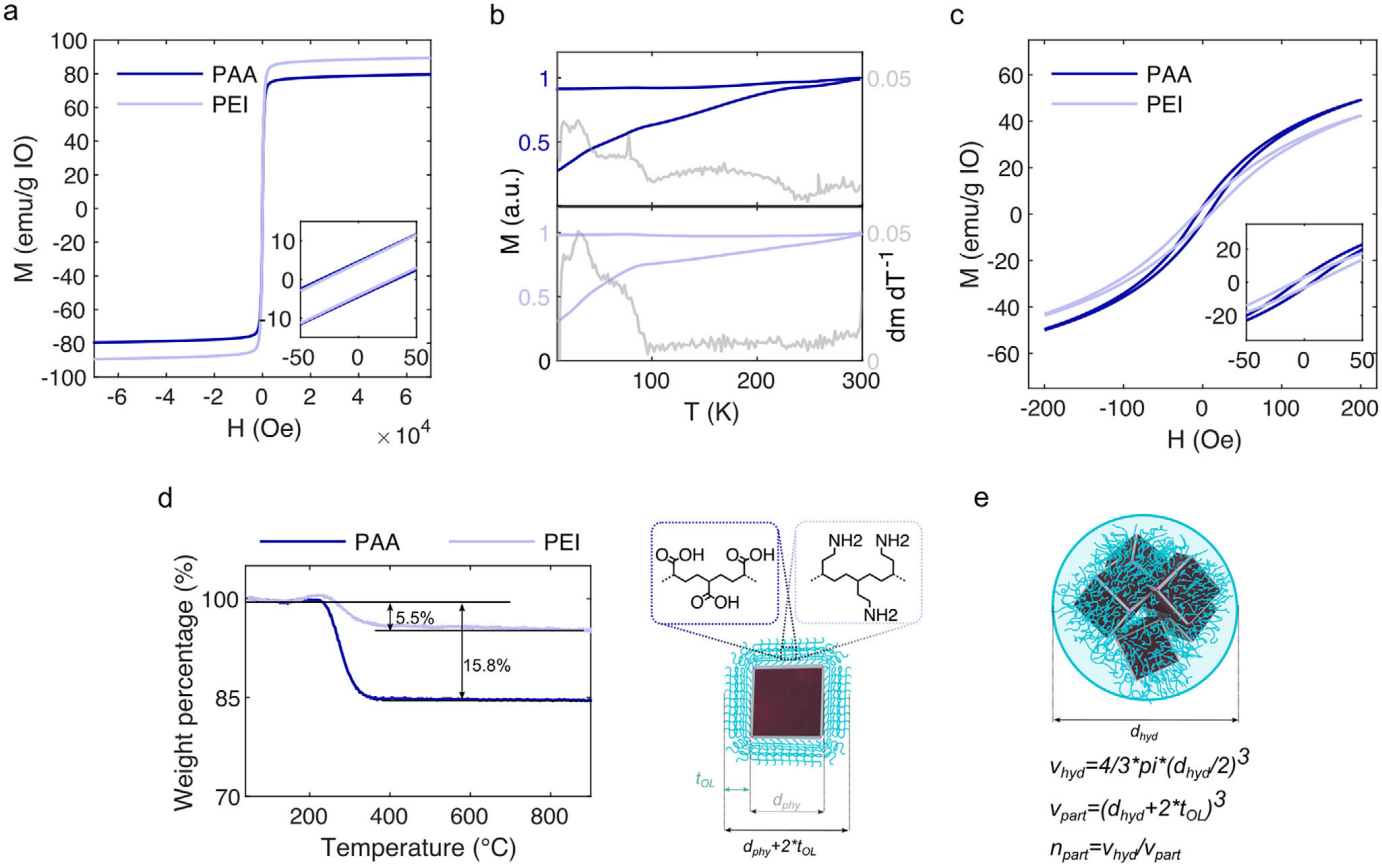
a) Hysteresis loops from compacted powders of functionalized MIONPs obtained at 300 K. b) ZFC‐FC curves and the derivatives of the ZFC magnetizations (grey lines) of compacted functionalized MIONP powders. c) Minor hysteresis loops at 300K obtained from freely arranged powders of functionalized MIONPs. d) Weight loss profiles of functionalized MIONPs between 30–900 °C obtained via TGA, and a schematic illustration of the organic surface layer thickness. e) Schematic illustration of the functionalized MIONPs hydrodynamic diameter and their derived aggregation state.

In order to better understand the origin of the observed different dynamic magnetic properties,^[^ ^26^ ^]^ we next conducted a detailed analysis of the physical properties of the particles in dry powder state. Powder XRD patterns of both functionalized nanoparticles showed a similar inverse spinal structure with an equal crystalline size of ≈15 nm (Figure S4a,b, Supporting Information). However, magnetic hysteresis loops recorded at 300 and 10 K performed in compacted dried particle powders unveiled a notably increased magnetic saturation for PEI‐functionalized particles (300 K: 90 emu g^−1^_IO_ (PEI), 80 emu g^−1^_IO_ (PAA); 10 K: 93 emu g^−1^_IO_ (PEI), 88 emu g^−1^_IO_ (PAA)) (**Figures** **3**a and S5, Supporting Information). Differences in saturation magnetization and in the coercive field value for the PAA (214 Oe) in comparison with PEI (180 Oe) might be related to an increased oxidation state of PAA‐functionalized particles,^[^ ^45^ ^]^ caused by the anchoring carboxylate groups. A close inspection of the zero‐field cooling and field cooling (ZFC‐FC) curves displayed in Figure 3b, supported our hypothesis, as the characteristic Verwey transition of magnetite observed at ≈90 K for PEI disappears in the PAA‐functionalized MIONPs. The loss of this transition indicates Fe^2+^ depletion and possibly an increased degree of structural disorder at the surface, both of which are consistent with stronger surface anisotropy contributions.^[^ ^45^, ^46^ ^]^ These effects can directly influence the heating efficiency under AC fields, as they modify the balance between Néel relaxation and hysteresis losses, thereby providing a plausible explanation for the differences in SAR observed between functionalized samples. The notable decrease in the Verwey transition temperature, compared to bulk material counterparts, was in line with previously reported cubic nanoparticles in this size range^[^ ^39^ ^]^ and can be substantiated by a size dependent transition from cubic‐to‐uniaxial crystalline symmetry within the particles.^[^ ^47^ ^]^ Furthermore, it is observed that the ZFC curve lacks a clear maximum. Additionally, the irreversibility point, where the ZFC and FC curves begin to diverge, is not present within the measured range. Therefore, the absence of a peak in the ZFC data, along with an almost constant FC magnetization value, suggests the presence of significant interparticle dipolar interactions. Figure 3c shows the minor loop hysteresis behavior of PAA and PEI‐functionalized MIONPs between −20 and 20 mT, which indicates on a slight low field magnetic susceptibility change of the MIONPs.

While the derived altered oxidation state may account for the changes observed in AC and DC hysteresis loops, we additionally conducted a detailed analysis of the particle aggregation state to gain insights into possible intra‐aggregate dipole interactions. An average ligand thickness (*t_OL_*) of ≈1.36 nm (PAA) and ≈0.64 nm (PEI) was calculated from the organic weight loss obtained from thermogravimetric analysis (TGA) of dried particle powders (Figure 3d) (Ligand densities were calculated according to Calculation C1, Supporting Information). We attribute the considerably lower ligand amount of PEI in comparison to PAA to the underlying branched structure of the employed PEI molecules, allowing for easier anchoring via multiple amino (–NH_2_) groups per molecule. Furthermore, the binding affinity of –NH_2_ groups toward the iron oxide surface is significantly weaker than that of carboxylate groups (–COOH) in PAA, which consequently reduces the occurring coupling rate. Assuming that the measured hydrodynamic diameter (d_hyd_) of the colloidal suspensions corresponds to the average aggregate diameter, we next approximated the number of particles per aggregate *n_part_* (conceptually illustrated in Figure 3e). Calculations revealed an increased intra‐aggregate particle packing amount *n_part,_* for PEI‐functionalized MIONPs, ≈306 particles per aggregate, while PAA functionalization resulted in ≈222. Such intra‐aggregate interactions are known to decrease the displayed AC hysteresis loop area, resulting in a decreased SAR,^[^ ^26^ ^]^ making a definitive attribution of the changed magnetic heating capacity between the particles toward changed oxidation states or altered dipole interaction strength unfeasible.

**Figure 4:**
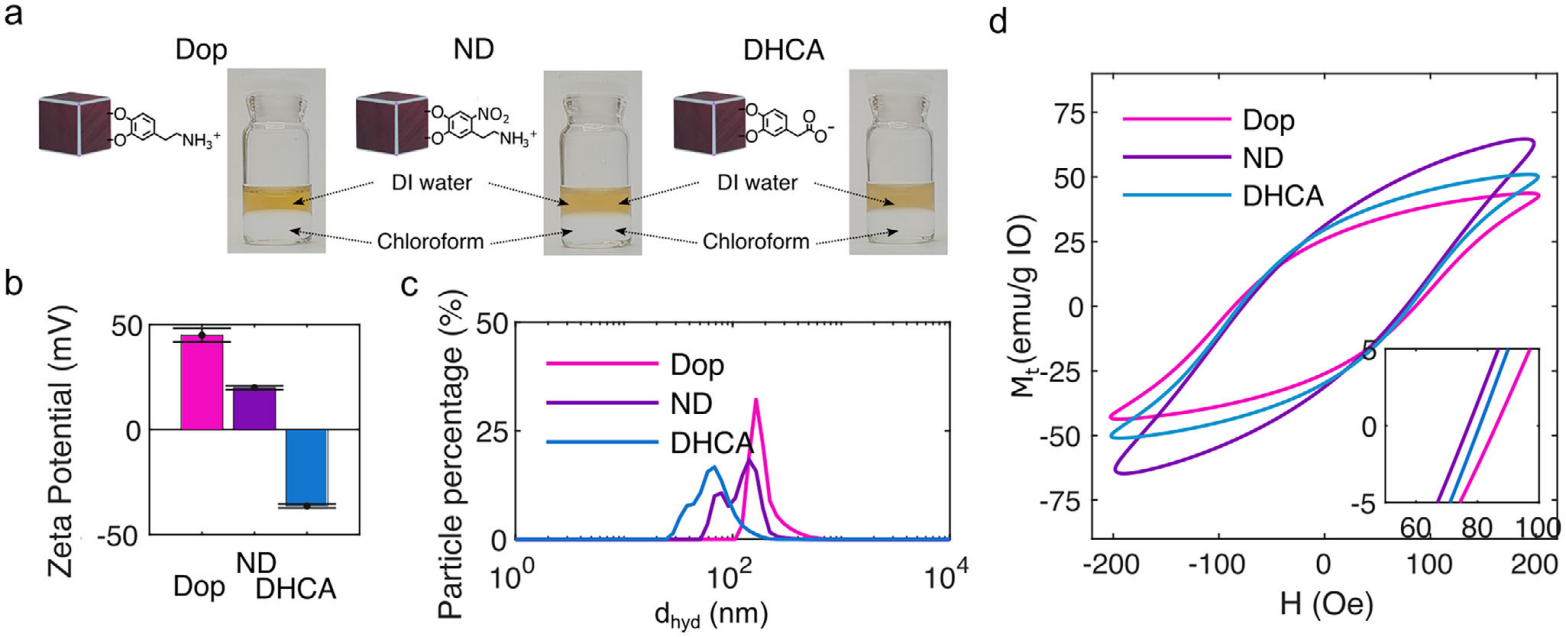
a) MIONPs in water on top of chloroform, after ligand exchanges. b) Measured ζ‐potentials of colloidal MIONP suspensions in de‐ionized water. c) Measured hydrodynamic diameter of diluted MIONP suspensions in de‐ionized water after functionalization. d) At 300K measured AC‐hysteresis loops of functionalized MIONPs in DI water.

Ionic stabilization via catechol‐based small molecules. Based on the previous observations, we proceeded next to functionalize the as‐synthesized OA‐capped particles with small molecules incorporating catechol as grafting anchoring groups, while varying their terminal groups, namely, dopamine (Dop), 3,4‐dihydroxyhydrocinnamic acid (DHCA), and 6‐nitrodopamine (ND), in order to gain a better understanding of the influence of surface ligands on the characteristic *SAR* of MIONPs. The high iron‐ion affinity of the selected molecules was chosen to ensure a high ligand grafting density via single molecular anchoring points on the particle surface, while simultaneously preventing the occurrence of undesired surface reactions, i.e., particle oxidation. As depicted in **Figure** **4**a, all the functionalization resulted in highly stable colloidal MIONP suspensions in water, which we attribute to the introduced positive and negative charges to the particle surface (Figure 4b). Dynamic light scattering (DLS) measurements of the MIONPs in aqueous solutions, however, revealed notable variations in their dispersion states, with average hydrodynamic diameters of 69 nm (DHCA), 125 nm (ND), and 192 nm (Dop) (Figure 4c).

To gain a better understanding on the changed intra‐aggregate dipolar interactions, we next conducted TGA measurements on dried particle powders (Figure S6, Supporting Information). The obtained profiles revealed a significantly increasing amount of organic material for ND‐ and Dop‐functionalized particles, with theoretical coupling densities of 2.6 (DHCA), 7.3 (ND), and 23.2 (Dop) molecules nm^−2^ (Calculated according to Calculation C2, Supporting Information). Considering a maximum of available binding sides on the particle surface of ≈2.8 Fe‐ions per nm^−2^, saturated Fe‐cation binding for all particle modifications must be assumed. This consequently results in different organic shell thickness according to DHCA (0.5 nm) < ND (1.3 nm) < Dop (3.1 nm), which can be likely attributed to nitrodopamine and dopamine polymerization during the ligand exchange process.^[^ ^48^ ^]^ Subsequent approximations of the average number of MIONPs within a formed aggregate (according to their hydrodynamic diameter), yielded interactions between ≈50 (DHCA), ≈219 (ND), and 443 (Dop) particles per aggregate, defining the strength of dipolar interactions between the samples according to DHCA < ND < Dop. Consequently, we hypothesized that investigations of the samples heating under magnetic AC stimulation would reveal significantly different heating capacities driven by intra‐aggregate dipole‐interactions. AC hysteresis loops, shown in Figure 4d, confirmed this hypothesis, giving *SAR* values of 589 W g^−1^ (Dop), 650 W g^−1^ (DHCA), and 754 W g^−1^ (ND). In contrast, the temperature profiles recorded during AC stimulation do not directly follow the heating trend derived from the AC hysteresis loop (Figure S7, Supporting Information), which can be attributed to the inherent limitations of calorimetric measurements and to variations in particle stability depending on the surface molecules present.^[^ ^49^, ^50^, ^51^, ^52^ ^]^

**Figure 5:**
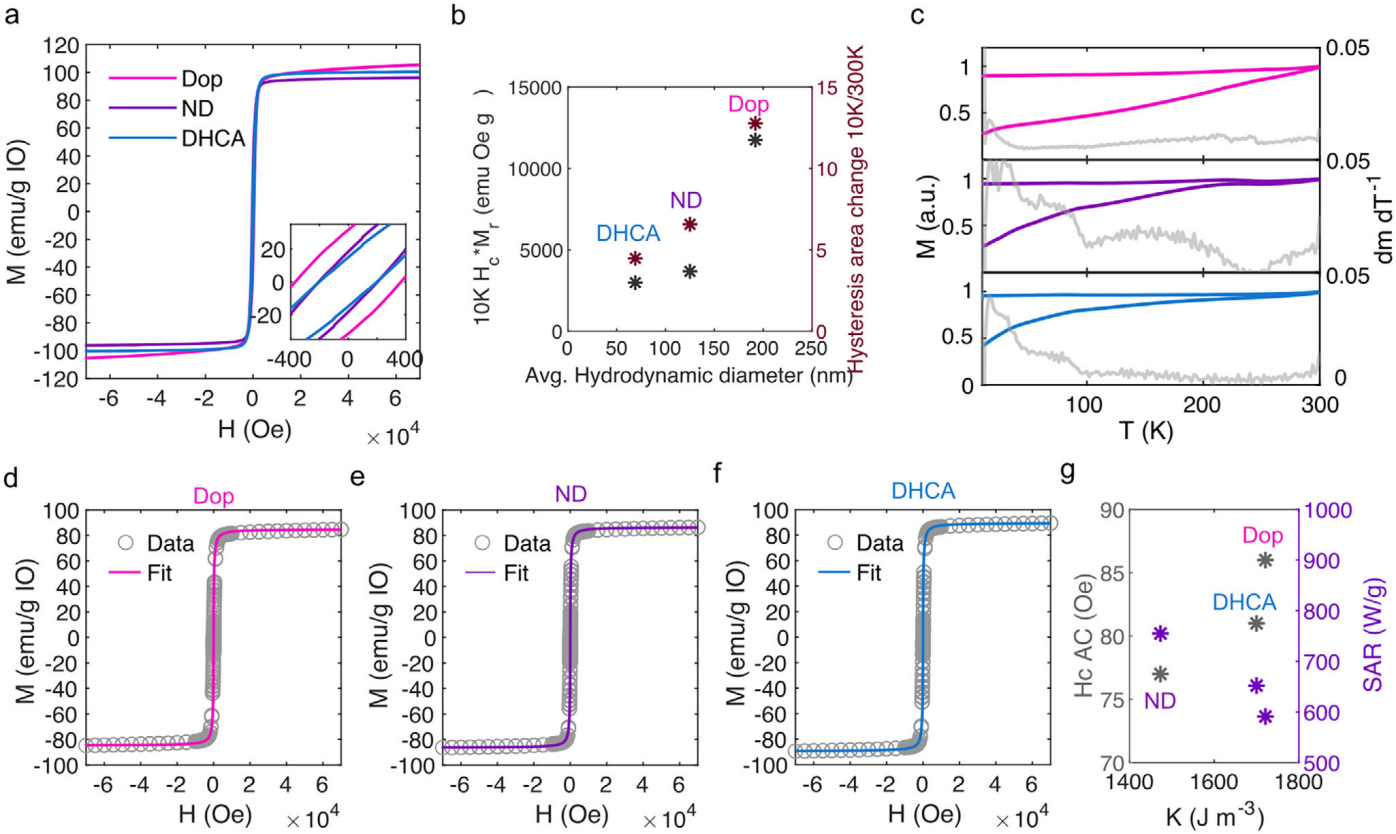
a) Hysteresis loops at 10K obtained from compacted powders of functionalized MIONPs. b) Illustration of the functionalized MIONPs at 10K measured Hc^*^Mr values and temperature dependent hysteresis area changes in dependency of their colloidal suspensions’ hydrodynamic diameters. c) ZFC‐FC curves and the derivatives of the ZFC magnetizations (pink DOP; violet ND; blue DHCA) of compacted functionalized MIONP powders. d–f) Hysteresis loops at 300K obtained from compacted powders (grey), and their respective hysteresis loop derived via Langevin fitting. g. Illustration of the functionalized Hc values obtained from AC hysteresis loops and their respective calculated SAR values in dependency of their calculated magnetic anisotropy.

Since the observed *SAR* trend for this series of samples does not appear to be completely linked to potential dipolar interactions, which would indicate on an order of DOP < ND < DHCA, we have conducted a detailed investigation of their static magnetic properties. The magnetic hysteresis loops recorded at 10 K depicted in **Figure** **5**a, revealed that also the hysteretic behavior shows a notably modified characteristics as a function of the catechol molecules, specifically the observed product of the MIONPs remanence (M_r_) and coercive field (H_c_) dependence seems to correlate with increasing aggregation (Figure 5b), confirming a changed magnetic response in dependency of the utilized ligand. Additionally, conducted measurements of the samples' ZFC‐FC curves, depicted in Figure 5c, revealed the vanishing of the Verwey transition, which was most pronounced for the dopamine functionalized MIONPs, indicating a similar surface‐binding‐induced oxidation change or increased structural disorder as previously observed for the PAA modified particles. However, all the samples present similar saturation magnetizations at 300 K, i.e., ≈87 ± 2 emu g^−1^_IO_, with similar minor loop behavior (Figures S8 and S9, Supporting Information), which points toward unchanged oxidation states between the catechol functionalized particles. Therefore, we hypothesized that a second surface‐related effect must be additionally affecting the particles' magnetic behavior at 300 K and, thus, their heating efficiency. We base this hypothesis on a previously reported ligand coupling type dependent surface electron depletion,^[^ ^43^ ^]^ which consequently must cause an altered electron spin‐orbit coupling among the MIONPs surface, thereby changing their inherent surface anisotropy. We therefore undertook quantifications of the MIONPs magnetic properties by calculating the MIONPs effective relaxation times τ using Linear Response Theory (LRT) to interpret their generated heat according to:^[^ ^19^, ^53^, ^54^ ^]^

(2) $$SAR=\frac{\pi \mu_{0}H^{2}f\chi_{0}}{\rho }\frac{2\pi f\tau }{1+{\left(2\pi f\tau \right)}^{2}}$$

Here, χ_0_ is the equilibrium or DC volume susceptibility:

(3) $$\chi_{0}=\frac{M_{S}\rho }{H}\left(\coth\left(\xi \right)-\frac{1}{\xi }\right)$$

(4) $$\xi =\frac{\mu_{0}V_{m}M_{S}\rho }{k_{B}T}H$$

where *H* and f is the magnetic field amplitude and frequency (200 Oe, 480 kHz), *M*_S_ the saturation magnetization of the particles (emu g^−1^), *T* the environment absolute temperature (in this case 300 K), *V_m_* the mean particles' magnetic volume, which was approximated for each functionalized sample from the Langevin function fitting (Figure 5d–f and Figure S10, Supporting Information) utilizing a log‐normal particle size distribution, ρ the magnetic material density (5 ×  10^6^ g m^−1^), *k*_B_ the Boltzmann constant, and μ_0_ the magnetic permeability of vacuum.^[^ ^55^, ^56^ ^]^ Afterward, we derived the particles' characteristic Néel and Brownian relaxation times before estimating their inherent effective magnetic anisotropy, K_eff_, assuming a characteristic attempt time τ_0,N_ of 10^−12^ sec and simplified expressions for both relaxation times:^[^ ^19^, ^57^, ^58^ ^]^

(5) $$\tau =\frac{\tau_{B}\cdot \tau_{N}}{\tau_{B}+\tau_{N}}$$

(6) $$\tau_{N}=\tau_{0,N}e^{\left(\frac{K_{eff}V_{\mathrm{mag}}}{k_{B}T}\right)}\;\tau_{B}=\frac{3\eta V_{H}}{k_{B}T}$$

Table 1 summarizes the measured, fitted, and calculated particle parameters. All samples exhibit similar magnetic volume, corresponding to a magnetic diameter ranging between 20 and 21 nm, in good agreement with the TEM diameters (Figure 2b). The equilibrium magnetic mass susceptibility values (χ_0_) are also similar for all functionalization. This suggests that the dipolar interactions induced by particle aggregation do not play an important role at room temperature magnetization processes (as also supported by the temperature dependence of the hysteresis area, depicted in Figure 5b). However, the effective relaxation time (τ) derived from the LRT is shorter for ND‐functionalized particles. Considering that for all ligands, the Brownian relaxation time τ_B_ is significantly larger than τ, it is evident that the dominant, and nearly exclusive, relaxation mechanism is the Néel relaxation, implying τ ≈ τ_N_. Therefore, the effective magnetic anisotropy constant (*K*_eff_) derived from Néel relaxation time (τ_N_) is 11% lower for ND functionalized particles. Consequently, no strong impact of the changed surface oxidation state or structural disorder for the other catechol‐ and PAA‐functionalized MIONPs, in comparison to the PEI‐functionalized particles, was derived. However, for the ND‐functionalized MIONPs the decrease in magnetic anisotropy showed to be in line with the decreasing magnetic coercivity in AC‐SQUID measurements, while simultaneously being inversely correlated with their measured *SAR* values (Figure 5g).

**Table 1:** Summary of measured and calculated particle parameters.

Functionalization	V_hyd_ [nm^3^]	Particles per aggregate	SAR [W g^−1^]	V_mag_ [nm^3^]	χ_0_[‐]	τ_*B*_[µs]	τ_*N*_[µs]	τ[µs]			*K_eff_* [J m^−3^]
DHCA	≈172·10^3^	≈50	650	4331	24,3	≈127		≈1,11	1,11	13.300	
ND	≈1022·10^3^	≈220	754	4741	24,6	≈754		≈0,92	0,92	12.000	
Dop	≈3706·10^3^	≈443	589	4252	24,3	≈2734		≈1,22	1,22	13.600	
PAA	≈1072·10^3^	≈222	631	4252	24,3	≈791	≈1,13	1,13			13.600
PEI	≈1098·10^3^	≈307	566	4252	24,3	≈810	≈1,28	1,28			13.700

We attribute the significantly reduced τ and K_eff_ for ND functionalized particles to the previously reported electron surface depletion associated with their nitrocatechol anchor group.^[^ ^43^ ^]^ This hypothesis was supported by a comparison of FTIR spectra obtained from dried particle powders and uncoupled ligand molecules (Figure S11a–c, Supporting Information). A close inspection of the in‐plane C─O stretch related vibrations ≈1280 cm^−1^ revealed a notable shift from 1285 to 1279 cm^−1^ for ND functionalized samples, pointing toward a strong electron delocalization from the surfaces Fe^2+^ ions. While only a weak signal could be obtained for DHCA functionalized particles, which is most likely caused by its lower coupling amount, no obvious peak shift was observed after particle functionalization. Moreover, DOP functionalization results in a slight C─O stretch vibration shift toward larger wavenumbers (1285–1288 cm^−1^), indicating an increased electron density at the particle surface, likely caused by the electron repulsion from the terminal amine moieties. Increased electron depletion from the particle surface after nitrodopamine functionalization was further supported by a pronounced shift toward higher wavenumbers for symmetric and asymmetric NO_2_ vibrations after coupling to the particle surface (sym: 1309–1321 cm^−1^; asym: 1539–1556 cm^−1^), confirming an increased electron density on the nitro group.^[^ ^43^, ^59^ ^]^

Considering the relatively large surface‐to‐volume ratio of the synthesized MIONPs, it can be estimated that ≈35% of the particles’ crystal unit cells are affected by surface‐related spin distortions. Such distortions are commonly linked to a reduction in the displayed magnetic susceptibility and saturation magnetization of MIONPs, caused by the presence of broken crystal structure symmetry and partially exposed ions at their surface, resulting in localized changes of their magnetocrystalline anisotropy.^[^ ^60^, ^61^, ^62^ ^]^ MIONPs are commonly described by a core–shell model, where the inorganic nanoparticle core is isolated from its environment by a surrounding layer of organic ligands. However, since the ligand anchoring group influences the electron orbital structure at the particle surface,^[^ ^43^ ^]^ electron spin–orbit coupling is expected to depend on both the anchoring type and grafting density. This suggests a direct link between the particle's magnetic surface anisotropy and its specific physicochemical surface design. Consistent with our results, a more accurate model of magnetic nanoparticles should incorporate a hybrid MIONP–ligand interface layer that reflects the altered electron orbital structure of iron ions coordinated to the ligand's anchoring groups, and their impact on the particle's magnetic surface spin states. Consequently, we conclude that the displayed magnetic heating capacity of MIONPs is governed by an intricate interplay between their inherent physical characteristics, surface design, and aggregation state.

**Figure 6:**
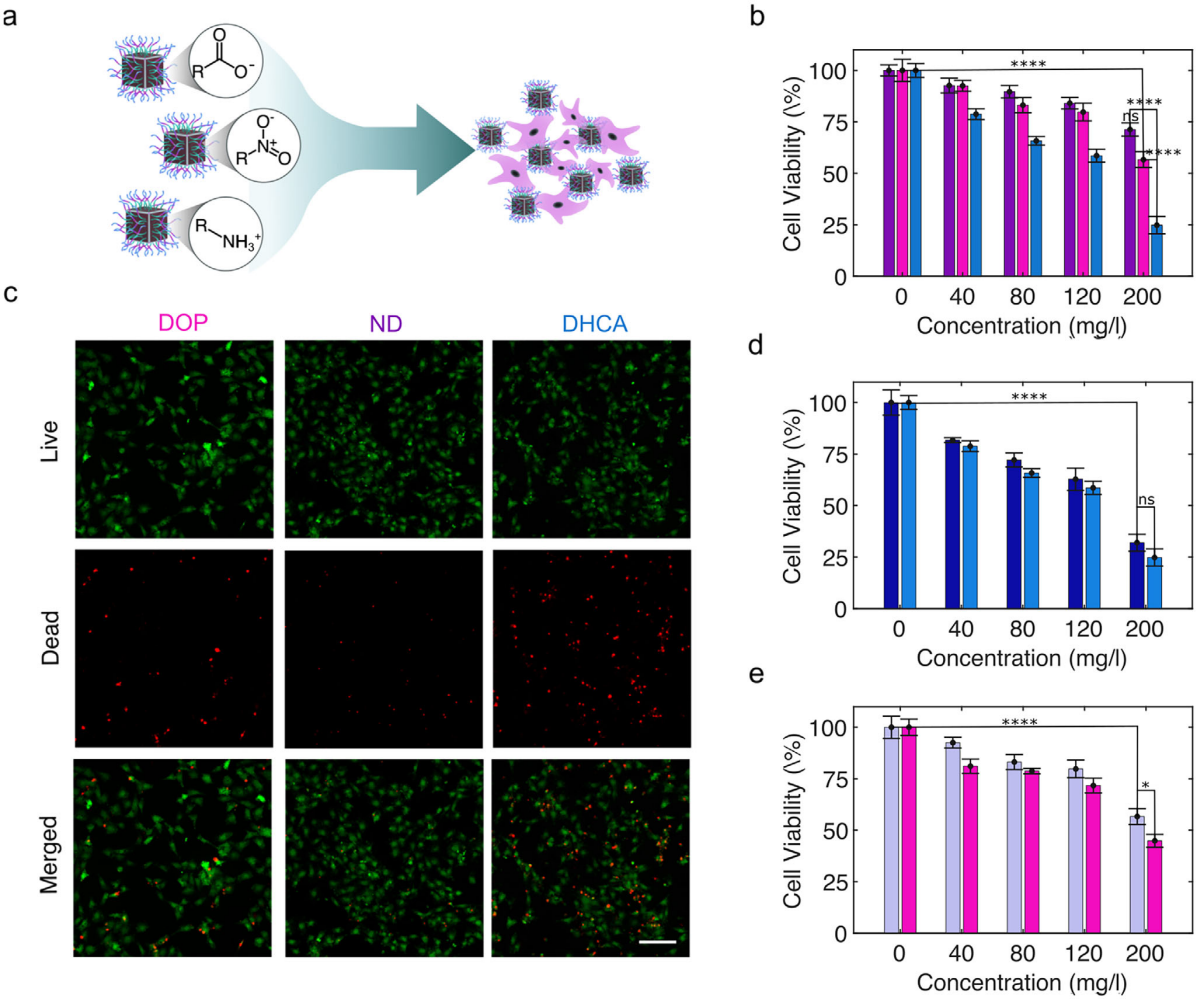
a) Schematic illustration of cell incubation with MIONPs functionalized with ligand molecules having different free chemical moieties on their surface. b) MTT assay with NIH/3T3 cells exposed to ND (violet), DOP (pink), and DHCA (light blue) functionalized MIONPs for 48 h. c) Live‐dead cell viability assay of NIH/3T3 cells exposed to nanoparticles functionalized with different ligand molecules after 48 h (scale bar: 200 µm). d) MTT assay with NIH/3T3 cells exposed to PAA (dark blue), and DHCA (light blue) functionalized MIONPs for 48 h. e) MTT assay with NIH/3T3 cells exposed to PEI (grey), and DOP (pink) functionalized MIONPs for 48 h. Statistical significance was calculated via one‐way ANOVA with a Tukey post‐hoc test (Figure 4c, d, f).^*^ *p* < 0.05, ^****^ *p*< 0.0001 versus control.

Assessment of cellular particle interactions. Researchers have increasingly focused on developing MIONPs for biomedical applications because of their superior biocompatibility compared to other magnetic nanoparticles. While this characteristic is commonly accepted, several studies have already shown that their displayed cytotoxicity is highly dependent on several physicochemical properties, such as their size, surface charge, and colloidal stability. Additionally, particles have been found to exhibit a varying toxicity depending on the exposed cell type, making extensive biocompatibility assessments essential for every MIONP type aimed toward biomedical deployment.^[^ ^32^, ^33^, ^63^, ^64^, ^65^, ^66^, ^67^, ^68^ ^]^ To investigate the impact of surface moieties on the particles’ biocompatibility, we next conducted in vitro evaluation of the functionalized MIONPs on embryonic mouse fibroblast cell line (NIH/3T3), as conceptually illustrated in **Figure** **6**a.

For all samples, MTT assays showed a decrease in cell viability with increasing particle concentration. Additionally, a ligand‐dependent effect was observed for catechol‐modified MIONPs (Figure 6b). The viability trend of exposed cells followed the order of ND > DOP > DHCA functionalization, which directly correlated with the results from the live‐dead assays (Figure 6c and Figure S12, Supporting Information). The observed trend, though, contradicts the commonly reported phenomenon of increased cytotoxicity for particles with positively charged surfaces.^[^ ^33^, ^69^ ^]^ Nevertheless, the significant decrease in cell viability for both DOP‐ and DHCA‐functionalized particles, compared to ND‐functionalized MIONPs, was consistent with the cell viability and live‐dead assay results obtained for PEI‐ and PAA‐functionalized particles. (Figure 6d,e and Figure S13, Supporting Information). We therefore conclude that the observed biocompatibility is directly influenced by the exposed chemical moieties (i.e. ‐NH_3_^+^ or ‐COO^−^). Furthermore, the observed poor cell viability in samples incubated with MIONPs with terminal carboxylic acid groups can likely be attributed to interactions with the cell membranes, potentially causing membrane damage, altered permeability, or hyperpolarization.^[^ ^70^, ^71^ ^]^ In contrast, particles functionalized with DOP and PEI induced a moderate cytotoxic response within cell lines, likely caused by their positive surface charges, which are known to enhance the cellular uptake of nanoparticles.^[^ ^72^ ^]^ ND‐functionalized MIONPs, however, displayed the lowest cytotoxicity while also displaying enhanced colloidal stability in physiological buffer solution (Figure S14, Supporting Information). We therefore conclude that the enhanced cell viability after exposure to ND‐functionalized particles is likely due to the capability of nitrodopamine to improve the colloidal stability of MIONPs within physiological conditions, thereby limiting direct particle‐cell membrane interactions. The presence of the nitro group in ND is known to enhance both the acidity and the particle's capacity to act as a hydrogen bond donor, potentially preventing oxidation of the catechol moieties and aggregation‐related cytotoxicity by simultaneously preventing salt bridge formation between the MIONPs.^[^ ^73^ ^]^

**Figure 7:**
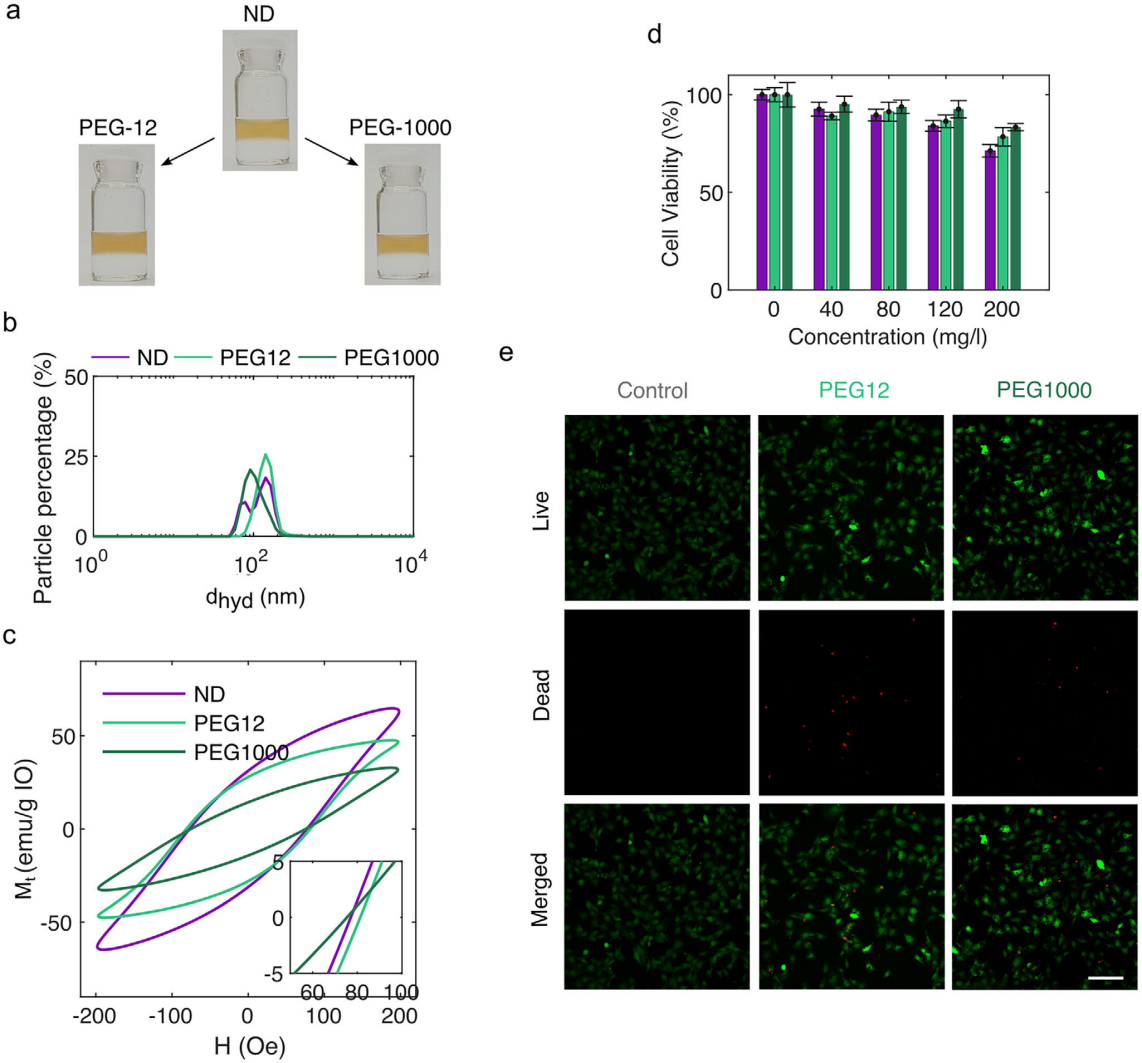
a) Nitrodopamine‐functionalized MIONPs in DI water before and after subsequent PEG coupling. b) Measured hydrodynamic diameter of diluted nitrodopamine‐functionalized MIONP suspensions in de‐ionized water before and after PEG coupling. c) AC Hysteresis loops of nitrodopamine functionalized MIONPs before and after PEG coupling. d) MTT assay with NIH/3T3 cells exposed to nitrodopamine (purple), nitrodopamine‐PEG12 (cyan), and nitrodopamine‐PEG1000 (green) functionalized MIONPs for 48 h witb one‐way anovo evaluation. e) Live‐dead cell viability assay of NIH/3T3 cells exposed to nanoparticles functionalized with different PEG12 and PEG1000 coupled nitrodopamine functionalized MIONPs (scale bar: 200 µm).

Steric hindrance‐based stabilization of ND functionalized MIONPs. Building on their favourable hysteresis losses, biocompatibility and colloidal stability, we undertook subsequent coupling of polyethylene glycol‐n‐hydroxysuccinimide (PEG‐NHS) ester chains with different molecular weights (PEG12 mw: 512 g mol^−1^; PEG1000 mw: 1000 g mol^−1^) to the ND‐functionalized particles according to a previously established protocol.^[^ ^74^ ^]^ As displayed in **Figure** **7**a,b, both couplings result in the formation of highly stable colloids having only slightly altered hydrodynamic diameter, while FTIR analysis of the dried particle powders after coupling revealed the PEG characteristic C─O related vibration peak at 1100 cm^−1^ thereby confirming successful conjugation of the molecules (Figure S15, Supporting Information). Magnetic AC hysteresis loop measurements of the PEG coupled MIONPs, unveiled a decreasing hysteresis area and, thus, in *SAR* with increasing PEG coupling length (≈20%: PEG12, ≈53%: PEG1000) (Figure 7c).

TGA on dried particle powders confirmed PEG‐grafting densities of ≈1.7 (PEG‐12) and ≈2.0 (PEG‐1000) molecules nm^−2^ (Figure S16, Supporting Information). We therefore conclude that the altered dynamic magnetic properties and, thus, their heating capacity of the PEG‐modified particles should be caused, solely, by dipole interactions between the PEG‐coated particle aggregates. The reduction of the AC susceptibility in the AC hysteresis cycles shown in Figure 7c, therefore suggests that dipolar interactions manifest in these minor loops as demagnetizing fields inside the aggregates. In contrast, the recorded heating profile showed a significant increase after coupling with short PEG chains, whereas coupling with higher molecular weight PEG led to a modest decrease in heating (Figure S17, Supporting Information). Although these variations are subject to the inherent limitations of calorimetric measurements, they may also point to friction‐related heating effects: PEG molecules in a brush conformation appear to enhance heating, while those in a mushroom conformation reduce it.

Lastly, we also conducted cell viability studies on the PEG‐coupled particles. MTT essay on cells exposed to the functionalized MIONPs for 48 h showed a decreasing cell viability with increasing particle concentration, which is in line with the previous observed trends for the investigated ligands (See Figure 7d). While no differences in ND‐ and PEG12‐functionalized MIONPs were observed, increased cell viability at higher concentration was observed for PEG1000 functionalized MIONPs. Additionally, live‐dead assays on the cells further confirmed this trend, as shown in Figure 7e and Figure S18 (Supporting Information). The observed increase in cell viability can be explained by enhanced steric repulsion between particles, resulting from the longer PEG chains providing greater surface coverage and preventing aggregation. Additionally, the neutral surface charge of PEG‐modified MIONPs promotes the formation of more stable colloidal suspensions in physiological buffer solutions while also reducing direct cellular interactions (Figure S19a,b, Supporting Information).

## 3 Conclusion

In this work, we investigated the influence of different surface molecules on the physicochemical, magnetic, and cytotoxic characteristics of MIONPs by varying the anchoring group, molecular weight, and end‐group of the surface ligands. The results revealed that the particles’ field‐dependent magnetization is directly influenced by their aggregation state and surface functionalization, which induce significant changes in the dipole–dipole interactions and surface anisotropy of the nanoparticle aggregates. Furthermore, it was shown that these changes have a notable impact on the MIONPs’ magnetic heating capacity, emphasizing the critical role of surface chemical modification for the future clinical translation of magnetic hyperthermia therapy. Cell viability assessments further demonstrated that negligible cytotoxicity can only be achieved when cells are exposed to functionalized particles lacking reactive terminal groups, generally requiring the presence of long, chemically inert polymer chains on the particle surface. Overall, the investigations presented contribute to advancing the understanding of the complex magnetic behavior of MIONPs and provide a useful reference for designing MIONP systems aimed at clinical magnetic hyperthermia applications.

## 4 Experimental Section

### Synthesis of MIONPs

Monodispersed MIONPs were synthesized according to a previously established protocol.^[^ ^44^ ^]^ In a typical synthesis iron acetylacetonate, (Thermo Fischer, CAS: 14024‐18‐1) (530 mg, 1.5 mmol) and sodium oleate (TCI, CAS: 143‐19‐1) (200 mg, 0.657 mmol) got dissolved in a mixture of 1‐octadecene (Sigma–Aldrich, CAS: 112‐88‐9) (15 mL), benzylether (Thermo‐Scientific, CAS: 103‐50‐4) (7 mL), 1‐tetradecene (Sigma–Aldrich: 1120‐36‐1) (3mL) and oleic acid (OA) (Sigma–Aldrich, CAS: 112‐80‐1) (1.6669 g, 5.907 mmol) via sonication. The slur was subsequently degassed for 60 min at 60 °C under vigorous stirring with an ellipsoid magnetic stirrer (2.0 x 1.0 mm; 1200 rpm), before it was heated to reflux at 294 °C under a N_2_ blanket with a heating rate of 3 °C min^−1^. After refluxing the solution for 90 min, the resulting crude product was allowed to cool to room temperature before it was collected and storing a particle containing stock solution at 4 °C for further processing.

### MIONP Purification

Before ligand exchange the as synthesized oleic acid covered MIONPs got purified by washing the crude product twice with a mixture of chloroform (Merck, CAS: 67‐66‐3) (25 mL) and acetone (Sigma–Aldrich, CAS: 67‐64‐1) (75 mL) via particle precipitation by centrifugation (14000 rpm) and follow‐up redispersion. Following, the collected particles were washed two more times by redispersion in chloroform (25 mL) and precipitation in a mixture of methanol (Sigma–Aldrich, CAS: 67‐56‐1) (50mL) and acetone (50 mL) via centrifugation. Finally, the purified oleic acid coated particles were dispersed in either tetrahydrofuran (THF) (Sigma–Aldrich, CAS: 109‐99‐9) or dry dimethylformamide (DMF) (Acros Organics, CAS: 68‐12‐8) in the desired concentration for further particle processing.

### Synthesis of Nitrodopamine Hydrogensulfate

Nitrodopamine hydrogensulfate was synthesized according to a previously reported protocol.^[^ ^74^ ^]^ In short, dopamine hydrochloride (Sigma–Aldrich, CAS: 62‐31‐7) (500 mg, 2.625 mmol) was dissolved in 15 mL de‐ionized water before adding sodium nitrate (Sigma–Aldrich, CAS: 7632‐00‐01) (6235 mg, 9.05 mmol) and cooling the mixture to 0 °C. Next, 5 mL of sulfuric acid (20% vol vol^−1^) (Sigma–Aldrich, CAS: 7664‐93‐9) was added dropwise to the mixture under vigorous stirring (ellipsoid magnetic stirrer 2.0 x 1.0 mm; 800 rpm) while maintaining the reaction flask in an ice‐bath. During the addition, the mixture underwent a color change from translucent to a muddy brown, followed by the formation of a yellow precipitate, which indicated on the formation of nitrodopamine hydrogen sulfate. After the reaction was complete, the mixture was stirred at room temperature overnight, before filtering the crude product and washing the collected nitrodopamine hydrogen sulfate 5 times with ice‐cold de‐ionized ‐water. Finally, the collected product was dried under a high vacuum before storing it at 4 °C for further use.

### Ligand‐Exchange of MIONPs with Polyacrylic Acid

Oleic acid coated MIONPs were functionalized with poly(acrylic acid) (PAA) via ligand exchange following a previously reported protocol with slight modifications.^[^ ^75^ ^]^ In a typical reaction, PAA (Sigma–Aldrich, CAS: 9003‐01‐4) (1.44 g, 0.72 mmol) was dissolved in 36 mL of THF under vigorous stirring (ellipsoid magnetic stirrer 2.0 x 1.0 mm; 800 rpm) in an inert atmosphere. Subsequently, 4 mL of a THF‐based nanoparticle suspension (10 mg mL^−1^) was added dropwise to the solution. Next, the mixture was incubated under a N_2_ blanked for 72 h while maintaining constant stirring. Finally, the PAA‐functionalized MIONPs got washed 5 times by sequential centrifugation (14000 rpm) and re‐dispersion in de‐ionized water before deprotonating them by the addition of 20 µl of NaOH (0.5 M) (Sigma Aldrich, CAS: 1310‐73‐2) and washing them another 3 times with de‐ionized water.

### Ligand‐Exchange of MIONPs with Polyethylenimine

Functionalization of oleic acid coated MIONPs with polyethylenimine (PEI) was undertaken by ligand exchange in THF. In short, branched PEI (1.44, 0.72 mmol) (Polysciences, CAS: 9002‐98‐6) was dissolved in de‐oxygenated de‐ionized water (4 mL) before adding THF (32 mL) under vigorous stirring (ellipsoid magnetic stirrer 2.0 x 1.0 mm; 800 rpm) under an inert atmosphere. Subsequently, 4 mL of THF containing MIONPs (10 mg mL^−1^) were added dropwise, and the mixture was stirred at room temperature under a nitrogen blanket for 72 h. The resulting PEI‐functionalized MIONPs were then washed five times by sequential centrifugation (14000 rpm) and redispersion in deionized water before protonating them by the addition of 10 µl of HCl (1 m) (Sigma Aldrich, CAS: 7647‐01‐0) and washing them another 3 times with de‐ionized water.

### Ligand‐Exchange of MIONPs with 3,4‐Dihydroxyhydrocinnamic Acid

As‐synthesized, oleic acid‐coated MIONPs were functionalized with 3,4‐dihydroxyhydrocinnamic acid (DHCA) using a well‐established ligand exchange protocol.^[^ ^76^ ^]^ In short, DHCA (Sigma–Aldrich, CAS: 1078‐61‐1) (150 mg, 0.82 mmol) was dissolved in THF by vigorous stirring (ellipsoid magnetic stirrer 2.0 x 1.0 mm; 800 rpm) under an inert atmosphere. The solution was subsequently heated to 50 °C, followed by the dropwise addition of 4 mL of a MIONP suspension in THF (10 mg mL^−1^). After addition, the mixture was allowed to react for 5 h, before it was cooled to room temperature and particles were washed 3 times by centrifugation (14000 rpm) and re‐dispersion in de‐ionized water. Finally, the DHCA‐functionalized particles were deprotonated by the addition of 10 µl of NaOH (0.5 M) and washed another 3 times with de‐ionized water to achieve a neutral pH dispersion.

### Ligand‐Exchange of MIONPs with Dopamine

As synthesized, oleic acid‐coated MIONPs were functionalized with dopamine (Dop) following a previously established ligand exchange protocol. Dopamine hydrochloride (Sigma–Aldrich, CAS: 62‐31‐7) (150 mg, 0.79 mmol) was first dissolved in 1 mL de‐oxygenated de‐ionized water before adding THF (15 mL) under vigorous stirring (ellipsoid magnetic stirrer 2.0 x 1.0 mm; 800 rpm). Subsequently, the solution was heated to 50 °C under an inert, before adding 4mL of MIONP suspended in THF (10 mg mL^−1^) dropwise and letting the mixture react for 5 h. Afterward, the solution was allowed to cool to room temperature, and the dopamine functionalized MIONPs were washed 3 times by centrifugation (14000 rpm) and re‐dispersion in de‐ionized water.

### Ligand‐Exchange of MIONPs with Nitrodopamine

Functionalization of oleic acid‐coated MIONPs with nitrodopamine (ND) was performed via ligand exchange according to a previously published protocol with minor adjustments.^[^ ^74^ ^]^ In a typical reaction, 120 mg of MIONPs were first dispersed in 30 mL of dry DMF under N_2_ bubbling. Subsequently, 40 mg (0.136 mmol) of the synthesized nitrodopamine hydrogensulfate was added to the dispersion while constantly bubbling N_2_ through the suspension. After 10 min of N_2_ purging, the mixture was sonicated for 60 min in an ultrasonic bath and then allowed to react at room temperature for 24 h. Afterward, the mixture as sonicated for another 60 min, before precipitating the nitrodopamine functionalized MIONPs in 300 mL of cold acetone. Subsequently, the functionalized particles were washed 5 times by centrifugation (14000 rpm) and re‐dispersion in methanol, before washing the MIONPs two more times in de‐oxygenated de‐ionized water following the same procedure before collection.

### Coupling of Nitrodopamine Functionalized MIONPs with Polyethylene Gylcol Derivatives

Nitrodopamine‐functionalized particles were coupled to polyethylene glycol (PEG) derivatives following a grafting to reaction as described in a previous report.^[^ ^74^ ^]^ In short, 40 mg of nitrodopamine‐functionalized MIONPs were dispersed in 4 mL dry DMF within a N_2_‐filled glove‐box. Following, the respective mPEG‐NHS ester derivative (200mg mPEG12‐NHS (PEG12)) (Broadpharm, CAS: 174569‐25‐6); 400mg mPEG‐NHS (mw: 1000 g mol^−1^) (PEG1000) (Biopharma PEG, CAS: MF001025‐1K) was added, and the mixture was allowed to react at room temperature for 16 h under continuous stirring. After reaction, the PEG coupled particles were precipitated in cold acetone (160 mL) and washed 5 times by centrifugation and re‐dispersion in de‐oxygenated de‐ionized water.

### Transmission Electron Microscopy (TEM)

TEM images were taken using a FEI Talos F200X (Chem S/TEM) operating at 200 kV, equipped with a X‐FEG emitter and CETA camera. Purified oleic acid‐coated particles were dispersed in chloroform and drop‐casted onto a carbon film‐coated copper grid for specimen preparation. Particle size distribution was determined by measuring the geometric parameters of 100 different MIONPs using FIJI ImageJ software.

### Hydrodynamic Diameter and Zeta‐Potential Measurements

Hydrodynamic diameters and zeta‐potentials of functionalized particles were taken using diluted dispersion either in de‐ionized water at 300 K or in phosphate buffered saline at 312 K. Measurements were performed either with a Malvern Panalytical Zetasizer Nano ZS, or an Anton Paar Litesizer 500 DLS.

### Magnetometry

The magnetic properties of the NPs were measured on compacted dried nanoparticles using a superconducting quantum interference device (SQUID) magnetometer (MPMS‐3, Quantum Design) up to 70 kOe equipped with a cryostat that can measure from 2 to 400 K. The magnetization versus applied field measurements were recorded both at room temperature (300 K) and at low temperature (10 K). The magnetization versus temperature measurements were carried out in ZFC and 50 Oe FC conditions.

Magnetic minor hysteresis loops of freely arranged MIONPs were measured using vibrating sample magnetometry (VSM) (Microsense EZ9) at 300 K, with an applied field range between −20 and 20mT. Samples were prepared by drop‐casting MIONPs suspended in de‐ionized water onto spherical filter paper substrates (∅ = 8*mm*), followed by drying under vacuum.

For all magnetic measurements, the MIONPs surface molecule related organic weight amount got determined by thermogravimetric analysis and used to approximate the weights of iron oxide (IO) present in each measurement, before calculating the particles' magnetization per gram iron oxide: *W_IO_* =  *W_Sample_* *%_*weight* *loss*_ and $M\left(emu/g\;IO\right)=\frac{M_{sample}}{W_{IO}}$, in which *W_Sample_* was was the total particle weight used in the magnetic measurement, %_*weight* *loss*_ the particle weight percentage remaining after TGA measurement, and *M_sample_* the magnetization of the sample was measured in the magnetic measurement.

### X‐Ray Diffraction (XRD)

The crystal structures of MIONPs were analyzed with a Malvern Panalytical Empyrean diffractometer equipped with a PIXcel detector and a copper X‐ray source (λ = 1.5406Å). Spectra were recorded over a 2θ range of 15°–80°, with a step size of 0.04°. Data processing, including noise reduction and baseline correction, was carried out with Profex. Crystallite size was calculated using the Scherrer Equation from the highest intensity peak after Gaussian fitting.

### AC Hysteresis and Specific Loss Power (SAR)

The AC hysteresis loops were measured by a lab‐made AC magnetometer, which produces a linear and homogeneous magnetic field across the sample volume.^[^ ^77^ ^]^ The AC magnetometer thereby used a pair of pick‐up and compensation coils, whereas the volume magnetization (in A/m) was numerically calibrated with additional VSM and calorimetric measurements. The mass magnetization (in emu/g) was obtained by normalizing the volume magnetization of the dispersion with the concentration of magnetic material. Afterward, the specific absorption rate, or SAR, was obtained from the hysteresis area by means of the following expression:

(7) $$SAR=-f\mu_{0}\int_{0}^{\frac{1}{f}}M_{t}dH$$

where *M_t_* was the mass‐normalized dynamic magnetization (in emu g^−1^), μ_0_ was the permeability of free space, whereas *f* and *H* were the applied magnetic field frequency and intensity (in Hz and A m^−1^). The integral runs over one AC magnetic field period, and it was calculated numerically after an FFT. All measurements were carried out at a thermal equilibrium at room temperature.

### Thermogravimetric Analysis (TGA)

The organic ligand amount on the particle surface was analysed by TGA performed on a Mettler Toledo TGA/DSC 3+ Star System. Particle dispersions in de‐ionized water were lyophilized, and the resulting dry powder was analyzed from 30–900 °C under a constant O_2_ flow (80 mL min^−1^).

### Fourier Transform Infrared Spectroscopy (FTIR)

FTIR spectra on lyophilized particle powders were obtained using a Varian 640 Fourier Transform Infrared Spectrometer equipped with golden gate‐diamond ATR. The spectra were recorded with 200 scans between 4000 – 400 cm^−1^ with a step size of 4 cm^−1^.

### NIH/3T3 Cell Culturing Methodology

The embryonic mouse fibroblast cell line, NIH/3T3, was cultured in a controlled environment using an incubator with 5% CO_2_ at 37 °C. The cells were maintained in Dulbecco's Modified Eagle Medium (DMEM, Gibco), enriched with 10% Fetal Bovine Serum (FBS) and 1% penicillin/streptomycin to prevent microbial contamination. The culture medium was replaced every two days, and the cells were passaged twice weekly.

### Cell Viability Assessment

Iron oxide nanoparticle samples were subjected to UV sterilization for 4 h. Following sterilization, NIH/3T3 cells were plated on 96‐well plates with a density of 2000 cells/well, using 0.2 mL DMEM. After allowing 24 h for cell adherence, the cultures were exposed to the sterilized iron oxide nanoparticle samples for 48 h. Following this exposure duration, 13 µl of sterilized MTT solution (3 mg mL^−1^ in Phosphate‐Buffered Saline, sourced from Thermo Fisher Scientific) was added to each well. Then the cultures were incubated at 37 °C for 4 h. After incubation, the culture medium was carefully aspirated from the wells. To ensure the solubilization of any remaining formazan crystals, 0.25 mL of dimethyl sulfoxide (DMSO) was introduced. The absorbance levels were assessed at 560 nm using an Infinite 200 PRO microplate reader (TECAN). Cell viability metrics, derived from these measurements, were represented as percentages, with values normalized against untreated control samples.

## Conflict of Interest

The authors declare no conflict of interest.

## Supporting information



Supporting Information

## Data Availability

The data that support the findings of this study are available from the corresponding author upon reasonable request.
