# Supplementary material for: Shaping Magnetic Hyperthermia Properties through Nanoparticle Surface‐Ligand Design: Implications for Cellular Responses
Source: Small. 2025 Oct 25;21(50):e07665. doi: 10.1002/smll.202507665 (PMC12710192; doi:10.1002/smll.202507665)
Supplement: Supplementary file 1 — Supporting Information [file SMLL-21-e07665-s001.docx]

Supporting Information

Shaping Magnetic Hyperthermia Properties Through Nanoparticle Surface-Ligand Design: Implications for Cellular Properties

*Lukas Hertle*, Alberto López-Ortega*, Hao Ye, Alba Martínez-Jiménez de Allo, Eneko Garaio, Valentin Gantenbein, Joaquim Llacer-Wintle, Ishika Paul, Sarina Nigg, Josep Puigmartí-Luis, Marta Estrader*, Xiang-Zhong Chen*, Bradley J. Nelson, Salvador Pané*


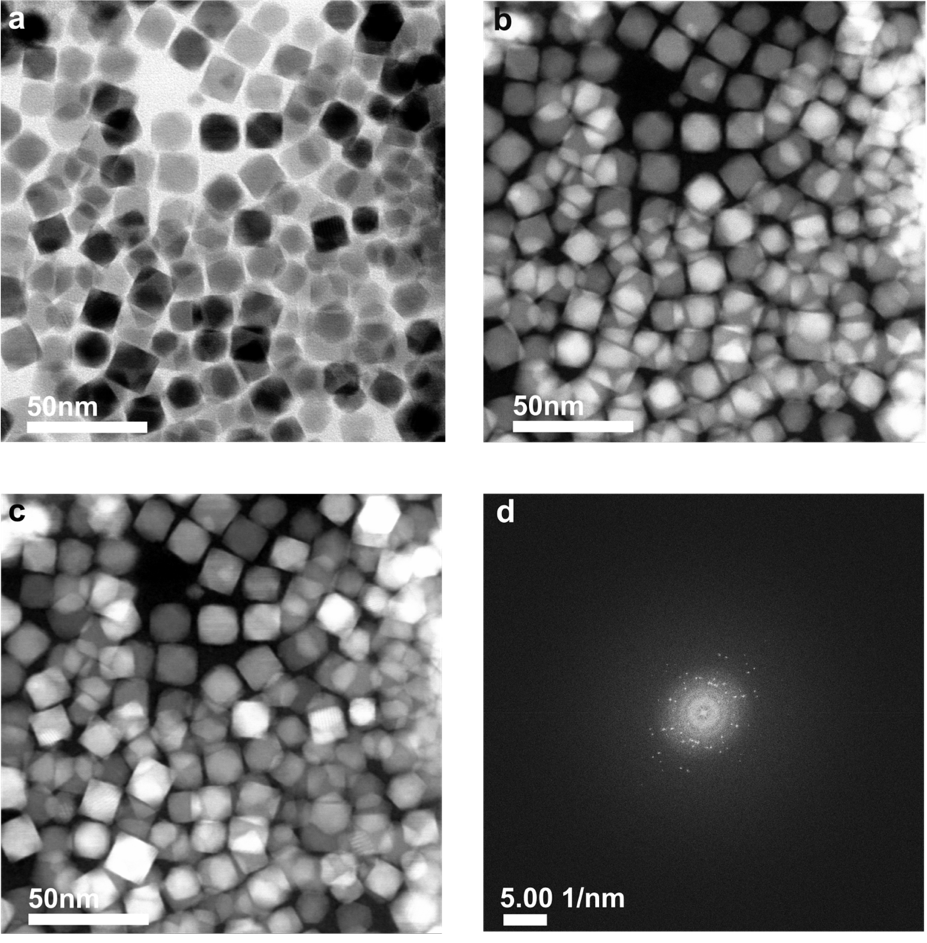


**Figure S1.** TEM images of as synthesized nanoparticles. **a**. Bright field. **b**. Dark field. **c**. HAADF image. **d**. Diffraction pattern


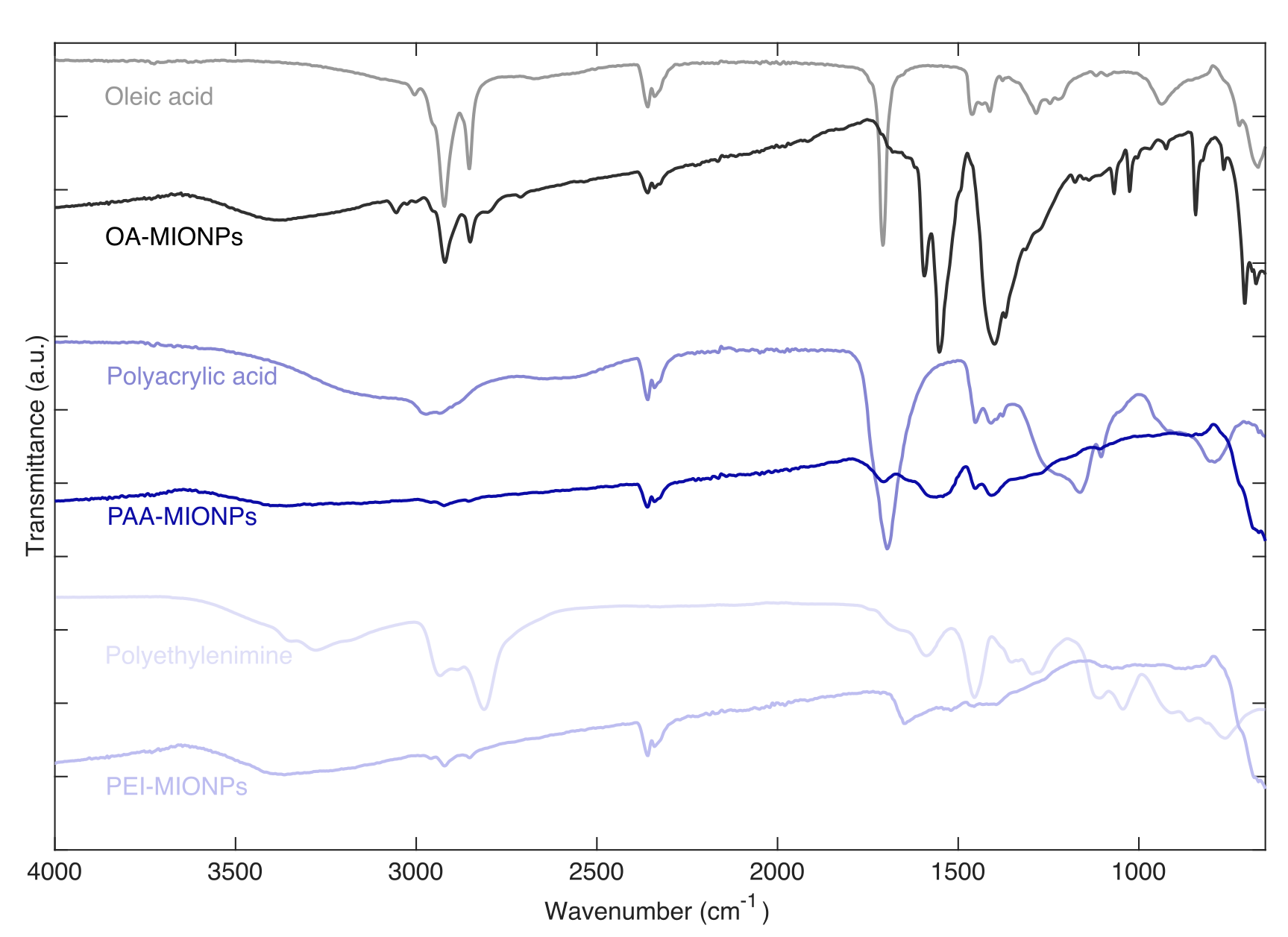


**Figure S2.** FTIR spectra of utilized polymer-based ligand molecules and their correspondingly functionalized MIONPs, as well as the spectra of oleic acid and as-synthesized MIONPs.


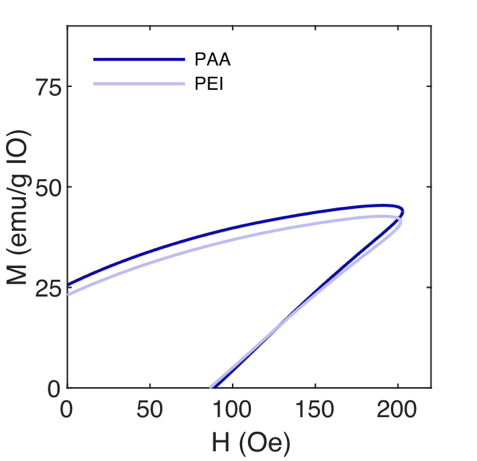


**Figure S3.** AC-hysteresis loop section recorded at 480 kHz and 200 Oe of field amplitude


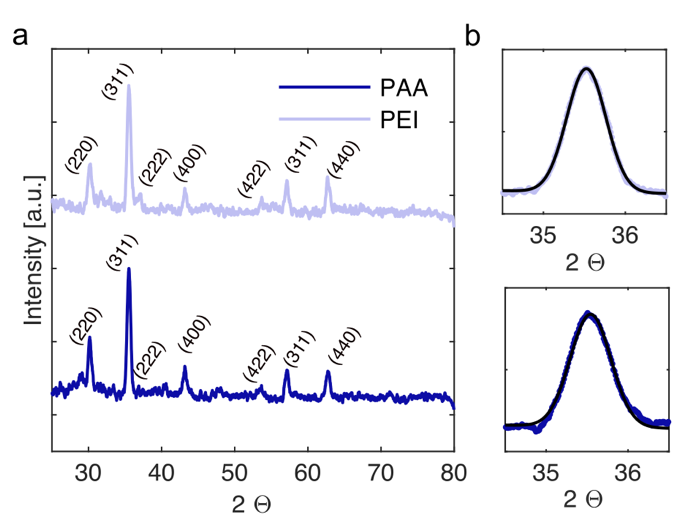


**Figure S4.** **a.** XRD spectra of dry nanoparticle powders functionalized with PAA and PEI. b. Gaussian fit vs measured and smoothed data on 311 peak.


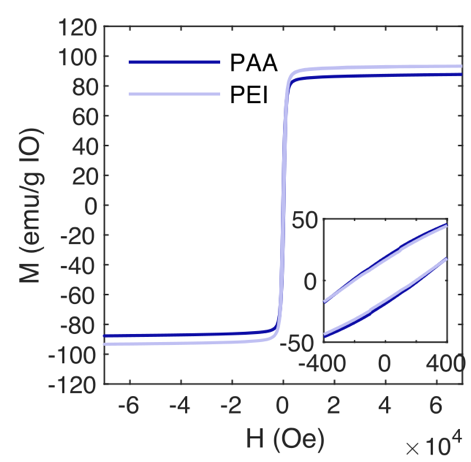


**Figure S5.** SQUID Magnetic hysteresis curves of compacted dry nanoparticle powders functionalized with different ligand molecules measured at 10K.

**Calculation C1:**

$v_{phy} = H_{TEM}*\left( \sqrt{\frac{{D_{TEM}}^{2}}{2}} \right)^{2}$; $w_{OL}=\frac{v_{phy}*\rho_{IO}}{{wl}_{tga}}-(v_{phy}*\rho_{IO})$

$d_{particle}=\left( \frac{w_{OL}}{\rho_{m}}+v_{phy} \right)^{\frac{1}{3}}$; $t_{OL}=\frac{d_{particle}-\left( v_{phy} \right)^{\frac{1}{3}}}{2}$

In which $v_{phy}$ refers to the particles physical volume, $H_{TEM}$ to the mean particle height determined from TEM, $D_{TEM}$ to the mean particle diagonal determined from TEM, $w_{OL}$ to the approximated weight of the organic layer on each particle, $\rho_{IO}$to the density of iron oxide, ${wl}_{tga}$to the particle weight percentage left after TGA measurements, $d_{particle}$ to the approximated particle edge length including the organic layer around the particles, and $t_{OL}$ to the approximated average ligand thickness present on the particles after coupling.


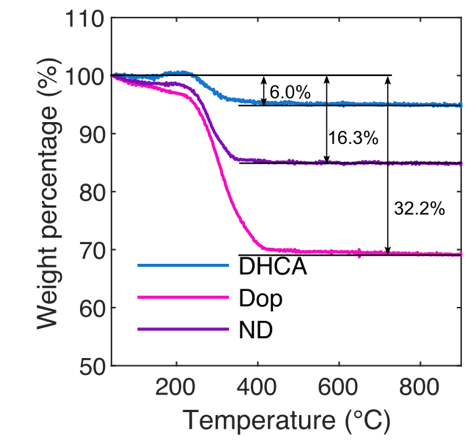


**Figure S6.** TGA curves of dry nanoparticle powders with different catechol-based ligands.

**Calculation C2:**

$v_{\mathrm{phy}} = H_{\mathrm{TEM}}*\left( \sqrt{\frac{{D_{\mathrm{TEM}}}^{2}}{2}} \right)^{2}$; $w_{OL}=\frac{v_{phy}*\rho_{IO}}{{wl}_{tga}}-(v_{phy}*\rho_{IO})$

${sa}_{phy}= {6*v_{phy}}^{\frac{2}{3}}$; $d_{OL}=\frac{\frac{w_{OL}}{w_{m}}*N_{A}}{{sa}_{phy}}$

In which $v_{phy}$ refers to the particles physical volume, $H_{TEM}$ to the mean particle height determined from TEM, $D_{TEM}$ to the mean particle diagonal determined from TEM, $w_{OL}$ to the approximated weight of the organic layer on each particle, $\rho_{IO}$to the density of iron oxide, ${wl}_{tga}$to the particle weight percentage left after TGA measurements, ${sa}_{phy}$ to the approximated particles surface area, $d_{OL}$ to the approximated theoretical ligand coupling densities present on the particles after coupling, $w_{m}$ to the ligand molecules molecular weight, and $N_{A}$ to the Avogadro constant.


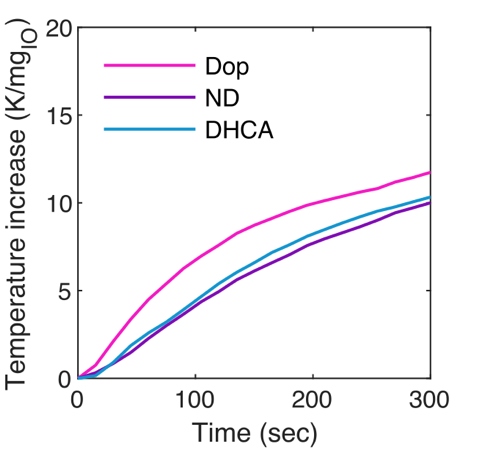


**Figure S7.** Recorded temperature change of colloidal MIONP suspensions of Dop, ND, and DHCA modified MIONPs, under 480 kHz and 200 Oe AC magnetic field, measured by fiber optic thermometry (Neoptix).


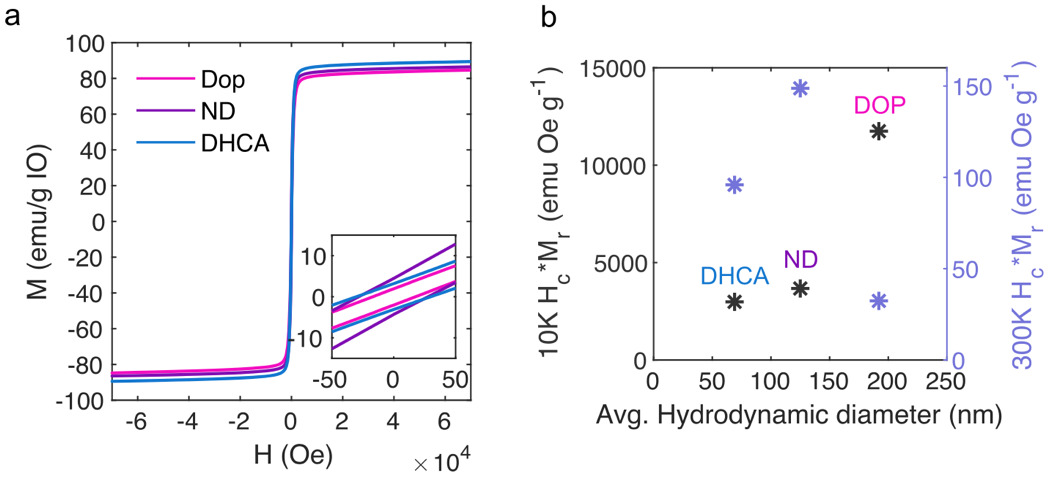


**Figure S8.** SQUID hysteresis curves measured on compacted samples of DHCA, dopamine, and nitrodopamine functionalized iron oxide nanoparticle at 300K.


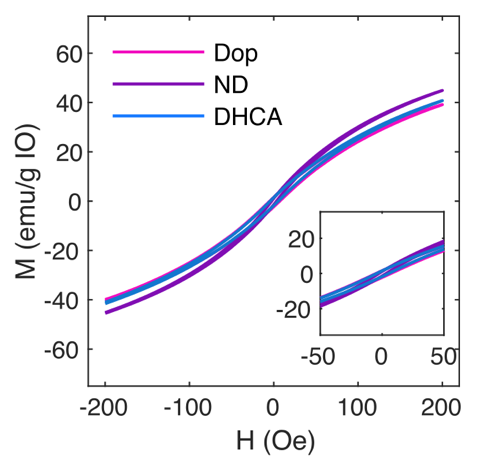


**Figure S9.** VSM measured minor hysteresis loops of freely arranged dried particle powders of MIONPs functionalized with different catechol-based ligand molecules.


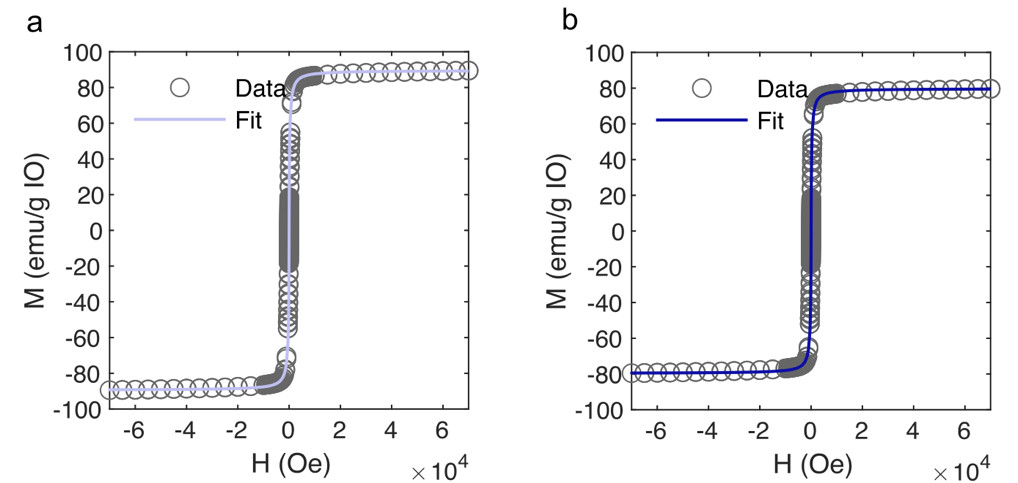


**Figure S10.** Langevin fitting on SQUID hysteresis curves measures at 300K of: **a.** PEI functionalized MIONPs. **b.** PAA functionalized MIONPs


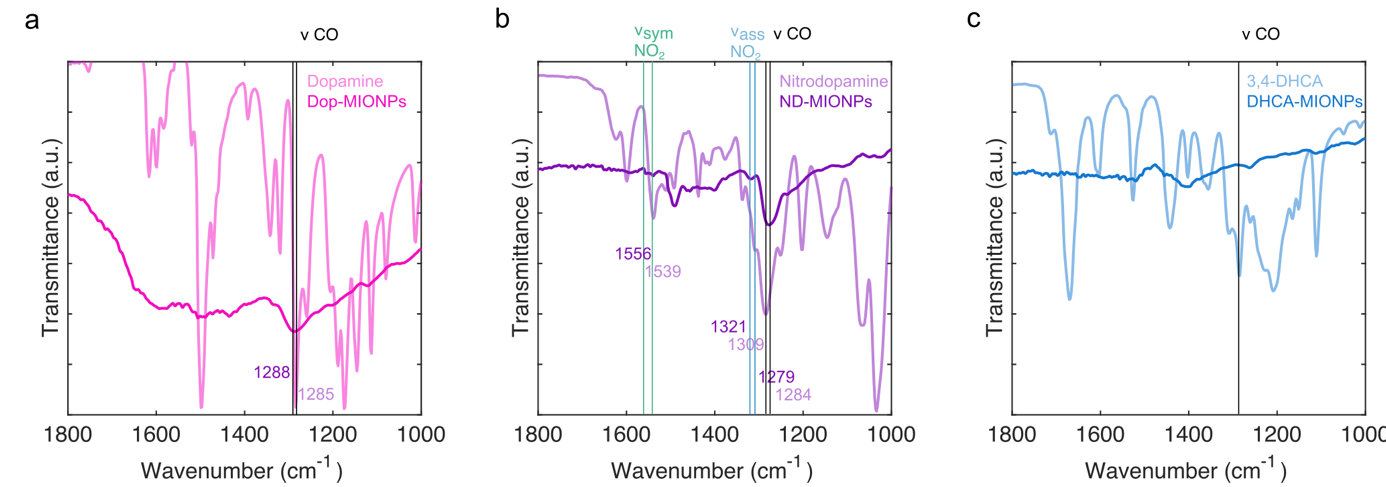


**Figure S11.** FTIR spectra of utilized catechol-based ligand molecules and their correspondingly functionalized MIONPs: **a.** Dopamine and Dop-MIONPs. **b.** Nitrodopamine and ND-MIONPs. **c.** 3,4-DHCA and DHCA-MIONPs


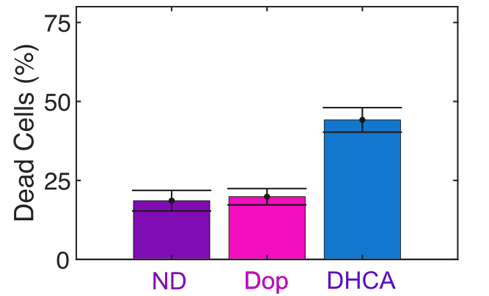


**Figure S12.** Live-dead assay of NIH/3T3 cells exposed for 48 hours to MIONPs functionalized with different catechol-based ligand molecules.


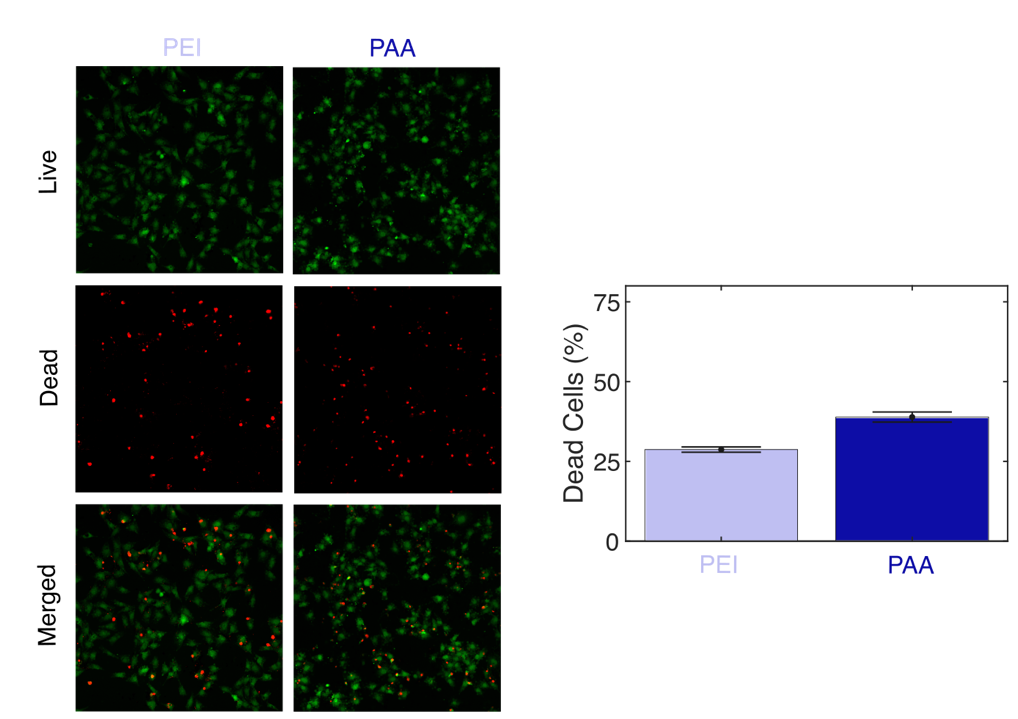


**Figure S13.** Live-dead cell viability assay of NIH/3T3 cells exposed to nanoparticles functionalized with different ligand molecules after 48 hours.

**
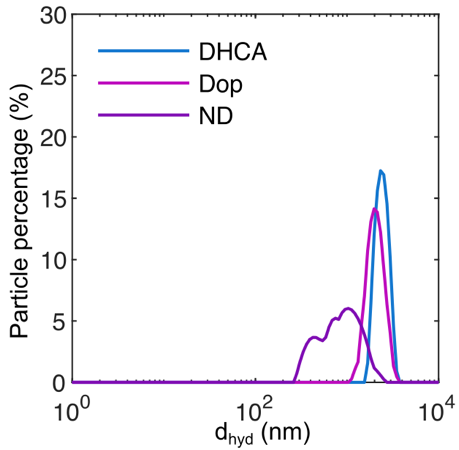
**

**Figure S14.** DLS determined, number weighted hydrodynamic diameters of colloidal dispersions of DHCA, dopamine, and nitrodopamine functionalized MIONPs in PBS.

**
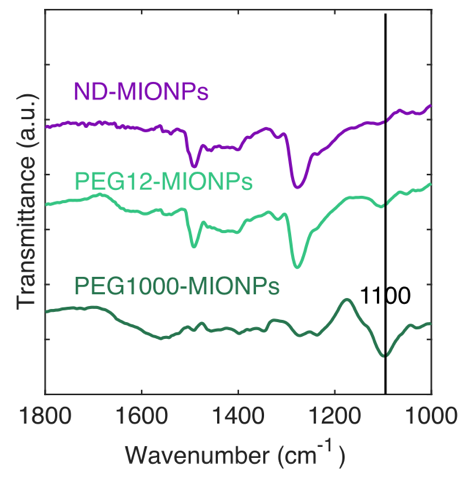
**

**Figure S15.** FTIR spectra of nitrodopamine-functionalized MIONPs before and after PEH coupling.


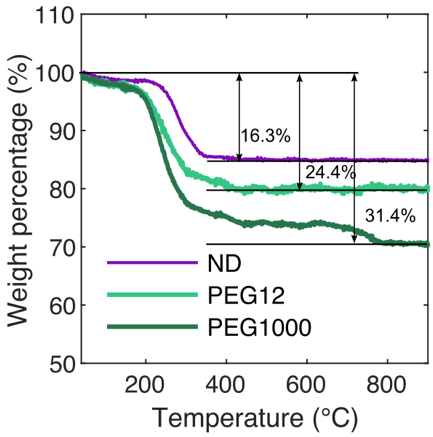


**Figure S16.** TGA curves of dry powders of ND functionalized, subsequently PEG12, and PEG1000 coupled MIONPs.


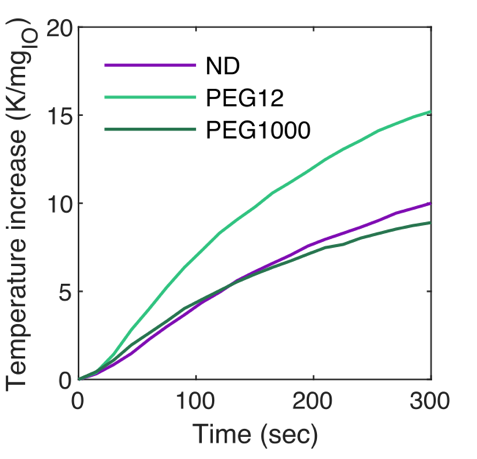


**Figure S17**. Recorded temperature change of colloidal MIONP suspensions of ND, functionalized MIONPs before and after PEG coupling, under 480 kHz and 200 Oe AC magnetic field, measured by fiber optic thermometry (Neoptix).


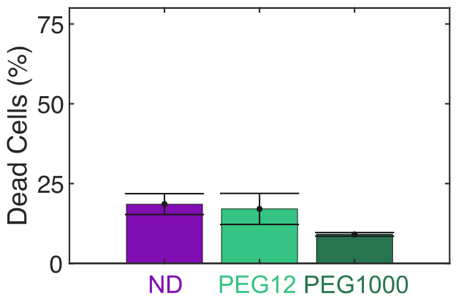


**Figure S18**. Live-dead cell viability assay of NIH/3T3 cells exposed to nanoparticles functionalized with different ligand molecules after 48 hours.


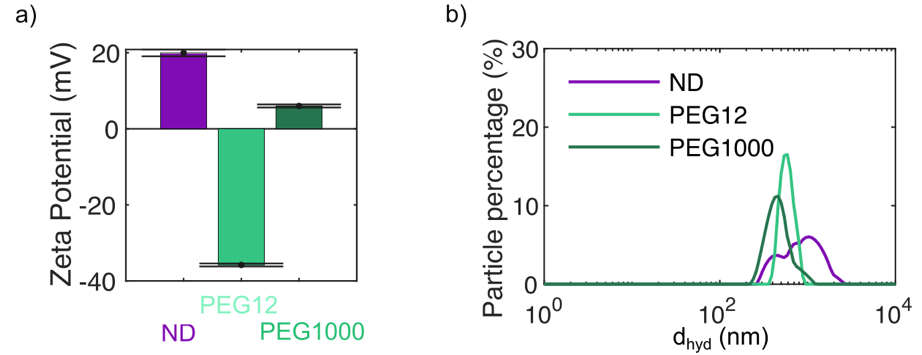


**Figure S19. a.** Zeta potentials of colloidal dispersions of ND, subsequently PEG12, and PEG1000 coupled MIONPs. **b.** DLS determined, number weighted hydrodynamic diameters of colloidal dispersions of ND-, subsequently PEG12-, and PEG1000- coupled iron oxide nanoparticle in PBS.
